# Design of New Benzo[*h*]chromene Derivatives: Antitumor Activities and Structure-Activity Relationships of the 2,3-Positions and Fused Rings at the 2,3-Positions

**DOI:** 10.3390/molecules22030479

**Published:** 2017-03-18

**Authors:** Rawda M. Okasha, Fawzia F. Alblewi, Tarek H. Afifi, Arshi Naqvi, Ahmed M. Fouda, Al-Anood M. Al-Dies, Ahmed M. El-Agrody

**Affiliations:** 1 Chemistry Department, Faculty of Science, Taibah University, 30002 Al-Madinah Al-Munawarah, Saudi Arabia; rawdao@yahoo.com (R.M.O.); ffs_chem334@hotmail.com (F.F.A.); afifith@yahoo.com (T.H.A.); arshi_84@yahoo.com (A.N.); 2 Chemistry Department, Faculty of Scinece, King Khalid University, P.O. Box 9004, 61413 Abha, Saudi Arabia; amfouda@hotmail.com (A.M.F.); nood-mansyaa@hotmail.com (A.-A.M.A.-D.); 3 Chemistry Department, Faculty of Science, Al-Azhar University, Nasr City 11884, Cairo, Egypt; elagrody_am@yahoo.com

**Keywords:** benzochromene, benzochromenopyrimidine, benzochromenotriazolopyrimidine antitumor activities, SAR study

## Abstract

A series of novel 4*H*-benzo[*h*]chromenes **4**, **6**–**11**, **13**, **14**; 7*H*-benzo[*h*]chromeno[2,3-*d*]pyrimidines **15**–**18**, **20**, and 14*H*-benzo[*h*]chromeno[3,2-*e*][1,2,4]triazolo[1,5-*c*]pyrimidine derivatives **19a**–**e**, **24** was prepared. The structures of the synthesized compounds were characterized on the basis of their spectral data. Some of the target compounds were examined for their antiproliferative activity against three cell lines; breast carcinoma (MCF-7), human colon carcinoma (HCT-116) and hepatocellular carcinoma (HepG-2). The cytotoxic behavior has been tested using MTT assay and the inhibitory activity was referenced to three standard anticancer drugs: vinblastine, colchicine and doxorubicin. The bioassays demonstrated that some of the new compounds exerted remarkable inhibitory effects as compared to the standard drugs on the growth of the three tested human tumor cell lines. The structure–activity relationships (SAR) study highlights that the antitumor activity of the target compounds was significantly affected by the lipophilicity of the substituent at 2- or 3- and fused rings at the 2,3-positions.

## 1. Introduction

In cancer research, multidrug resistance (MDR) is one of the major aspects that causes failure in therapeutic treatment. This phenomenon could occur either via inherited or acquired approaches, which refers to the initial resistance to a specific drug and the development of resistance after successful treatment, respectively. There have been numerous attempts to overcome these obstacles, including applying drug treatment in combination protocols. In the meantime, the development of new materials for drug design continues to be crucial in addressing this phenomenon.

Heterocyclic compounds, in particular oxygen-containing molecules, represent an indispensable class due to their physicochemical properties. The literature reveals that chromenes and benzochromenes are important pharmacophores associated with a broad range of pharmacological activities, such as antimicrobial [1,2,3,4], anticancer agent [5] hypolipidemic [6], antioxidant [7,8], analgesic [9], antileishmanial [10,11], vascular-disrupting activity [12], estrogenic anticoagulant and antispasmolytic [8], and blood platelet antiaggregating [13] effects.

Benzochromene compounds have been synthesized using various strategies such as multicomponent reactions (MCRs) [14,15,16,17], heterogeneous catalytic methods [18,19], electrocatalytic processes [20], microwave [21,22,23,24] and ultrasound techniques [25]. Multicomponent reactions are some of the most successful procedures to prepare this class of molecules in good yields. Meanwhile, microwave-assisted organic synthesis (MAOS) has been known for accelerating organic reactions, increasing product purities and simplifying the work-up [21,22,23,24]. Cyclocondensation reactions in “dry media” leading to heterocyclic systems have been performed under microwave irradiation [26,27,28,29,30,31], which were carried out in a neat, solvent-free state or in ethanol under microwave irradiation and helped to generate products not attainable through classical heating methods.

**Figure 1:**
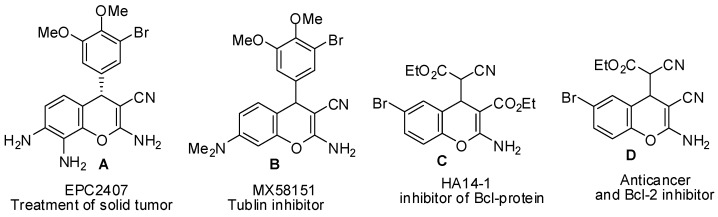
Structures of 2-amino-4*H*-chromenes with diverse biological and pharmacological activities.

Benzochromenes play a unique role in drug discovery programs. Furthermore, chromene derivatives are an important class of heterocyclic compounds that show a wide range of potent antitumor activity. For example, Crolibulin (**A**) is currently in Phase I/II clinical trials for the treatment of advanced solid tumors [32], 2-amino-4-(3-bromo-4,5-dimethoxyphenyl)-7-(dimethyl-amino)-4*H*-chromene-3-carbonitrile (**B**) has been known for tubulin inhibitor [33], while ethyl 2-amino-6-bromo-4-(1-cyano-2-ethoxy-2-oxoethyl)-4*H*-chromene-3-carboxylate (**C**) and 2-amino-6-bromo-4-(1-cyano-2-ethoxy-2-oxoethyl)-4*H*-chromene-3-carbonitrile (**D**) act as inhibitors of Bcl-2 protein and as an apoptosis inducer [34,35], respectively (Figure 1).

**Figure 2:**
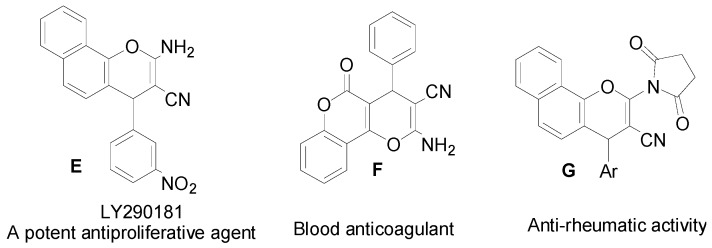
Structures of benzochromenes with diverse biological and pharmacological activities.

Other remarkable examples of benzchromenes have emerged in the treatment of human diseases. For instance, 2-amino-4-(3-nitrophenyl)-4*H*-benzo[*h*]chromene-3-carbonitrile (**E**) is a potent antiproliferative agent for a variety of cell types and inhibition of mitosis and microtubules [36,37], while 2-amino-5-oxo-4-phenyl-4,5-dihydropyrano[3,2-*c*]chromene-3-carbonitrile (**F**) serves as a blood anticoagulant analogue of warfarin [38]. Finally, 4-substituted-2-(*N*-succinimido)-4*H*-benzo[*h*]-chromene-3-carbonitriles (**G**) display anti-rheumatic activity [39] (Figure 2).

In view of these observations and as a continuation to our previous work on heterocyclic chromene moieties, we report the synthesis of new 4*H*-benzo[*h*]chromenes, 7*H*-benzo[*h*]chromeno-[2,3-*d*]pyrimidines and 14*H*-benzo[*h*]chromeno[3,2-*e*][1,2,4]triazolo[1–5-*c*]-pyrimidines. Some of the target compounds were examined for their antitumor activities in comparison to the standard drugs vinblastine, colchicine and doxorubicin. The structure–activity relationships of the desired molecules highlighted the effect of the substituents at the 2,3-positions and fused rings at the 2,3-positions on the antitumor activity.

## 2. Results and Discussion

### 2.1. Chemistry

**Scheme 1:**
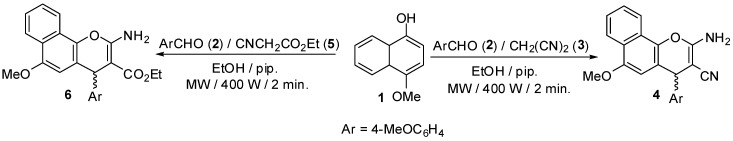
Synthesis of 4*H*-benzo[*h*]chromene derivatives **4** and **6**.

The 4*H*-benzo[*h*]chromene derivatives **4** and **6** described in this study were prepared according to the methodology illustrated in Scheme 1. The reaction proceeded via a one-pot three component condensation of 4-methoxy-1-naphthol (**1**), 4-methoxybenzaldehyde (**2**) and malononitrile (**3**) or ethyl cyanoacetate (**5**) in an ethanolic piperidine-containing solution under microwave irradiation for 2 min at 140 °C to afford the target compounds 2-amino-4-(4-methoxyphenyl)-6-methoxy-4*H*-benzo[*h*]chromene-3-carbonitrile (**4**) and ethyl 2-amino-4-(4-methoxyphenyl)-6-methoxy-4*H*-benzo- [*h*]chromene-3-carboxylate (**6**), respectively. The optical activities of the target compounds **4** and **6** were measured using a Carl Zeiss polarimeter. The results indicated that compounds **4** and **6** have zero rotation (i.e., they are optically inactive) and thus are in the form of a racemic (±) mixture as illustrated in Scheme 1.

**Scheme 2:**
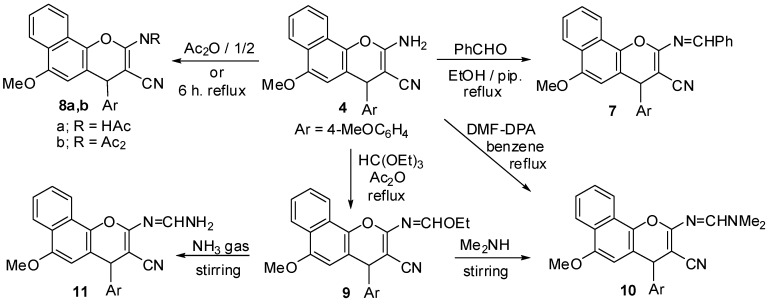
Synthesis of 2-substituted 4*H*-benzo[*h*]chromene-3-carbonitriles **7**–**11**.

Condensation of 2-amino-4-(4-methoxyphenyl)-6-methoxy-4*H*-benzo[*h*]chromene-3-carbonitrile (**4**) with benzaldehyde in ethanolic piperidine solution under reflux afforded the Schiff base product **7**. Acylation of compound **4** with acetic anhydride under reflux for 1/2 h gave the *N*-acetyl derivative 2-acetylamino-4-(4-methoxyphenyl)-6-methoxy-4*H*-benzo[*h*]chromene-3-carbonitrile (**8a**), while applying the same conditions for 6 h gave the corresponding *N*,*N*-diacetyl derivative 2-diacetylamino-4-(4-methoxyphenyl)-6-methoxy-4*H*-benzo[*h*]chromene-3-carbonitrile (**8b**). Furthermore, treatment of **4** with triethyl orthoformate in acetic anhydride or dimethylformamide-dipentylacetal (DMF-DPA) in benzene under reflux gave the corresponding desired products 2-ethoxymethyleneamino-4-(4-methoxyphenyl)-6-methoxy-4*H*-benzo[*h*]chromene-3-carbonitrile (**9**) and 2-dimethylaminomethyleneamino-4-(4-methoxy-phenyl)-6-methoxy-4*H*-benzo[*h*]chromene-3-carbonitrile (**10**), respectively. Reaction of the imidate **9** with dimethylamine in methanol at room temperature under stirring for 1 h yielded the imidine **10**, which can be obtained as described before from the reaction of **4** and (DMF-DPA), while ammonolysis of compound **9** with NH_3_ gas bubbled in methanol at room temperature under stirring for 1 h afforded the open chain product 2-aminomethyleneamino-4-(4-methoxyphenyl)-6-methoxy-4*H*-benzo[*h*]-chromene-3-carbonitrile (**11**). These reactions are depicted in Scheme 2.

**Scheme 3:**
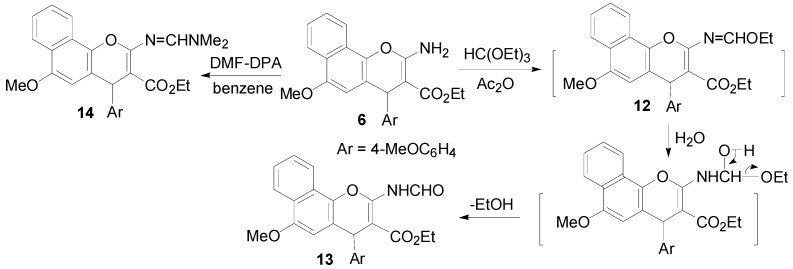
Synthesis of ethyl 2-formamido/dimethylaminomethyleneamino derivatives **13** and **14**.

Similarly, reaction of β-enaminoester **6** with triethyl orthoformate afforded ethyl 4-(4-methoxphenyl)-2-formamido-6-methoxy-4*H*-benzo[*h*]chromene-3-carboxylate (**13**), instead of the desired product, ethyl 2-ethoxymethyleneamino-4-(4-methoxphenyl)-6-methoxy-4*H*-benzo[*h*]-[*h*]chromene-3-carboxylate (**12**), while reaction of **6** with (DMF-DPA) produced ethyl 2-dimethyl-aminomethyleneamino-4-(4-methoxyphenyl)-6-methoxy-4*H*-benzo[*h*]chromene-3-carboxylate (**14**). The formation of compound **13** can be rationalized through initial formation of a product by adding H_2_O to the ethoxymethyleneamino group (-N=CHOEt) of **12**, which then loses ethanol to give the 2-formamido derivative **13** [40]. These results are depicted in Scheme 3.

**Scheme 4:**
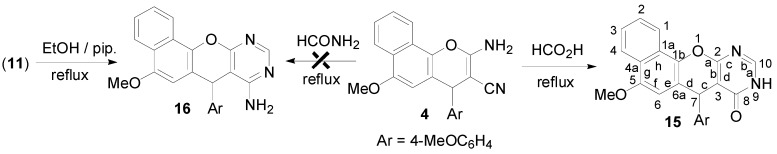
Synthesis of the pyrimidine derivatives **15** and **16**.

The β-enaminonitrile **4** and the imidate **9** proved to be useful precursors for the synthesis of a variety of pyrimidine derivatives. Thus, condensation of **4** with formic acid under reflux allowed for the formation of 7-(4-methoxyphenyl)-5-methoxy-7*H*,9*H*-benzo[*h*]chromeno[2,3-*d*]pyrimidin-8-one (**15**), while reaction of **4** with formamide under reflux was unsuccessful, as 8-amino-7-(4-methoxy-phenyl)-5-methoxy-7*H*-benzo[*h*]chromeno[2,3-*d*]pyrimidine (**16**) was not formed. In contrast, compound **16** could be synthesized by cyclization of **11** in ethanolic piperidine solution under reflux [41] (Scheme 4).

**Scheme 5:**
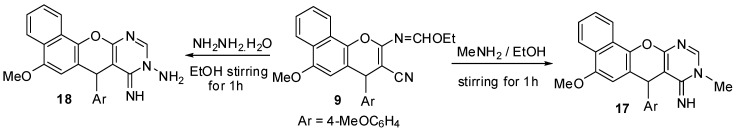
Synthesis of compounds **17** and **18**.

Interaction of the imidate **9** with methylamine in methanol at room temperature under stirring for 1 h afforded the cycloaddition product 7-(4-methoxyphenyl)-5-methoxy-8-imino-9-methyl-7*H*-benzo[*h*]chromeno[2,3-*d*]pyrimidine (**17**), while hydrazinolysis of **9** afforded the corresponding cycloaddition product 9-amino-7-(4-methoxyphenyl)-5-methoxy-8-imino-7*H*-benzo[*h*]chromeno-[2,3-*d*]pyrimidine (**18**). These results are depicted in Scheme 5.

**Scheme 6:**
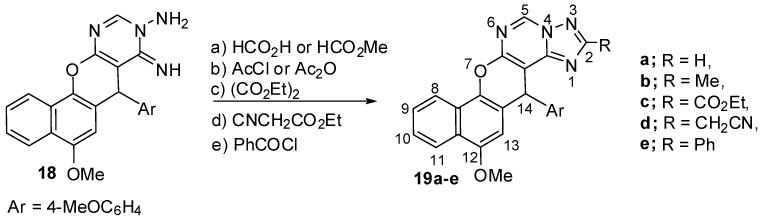
Synthesis of 2-substituted 14*H*-benzo[*h*]chromeno[3,2-*e*][1,2,4]triazolo[1,5-*c*]pyrimidines **19a**–**e**.

The aminoimino compound **18** proved to be a useful intermediate for the synthesis of a variety of 2-substituted 14*H*-benzo[*h*]chromeno[3,2-*e*][1,2,4]triazolo[1,5-*c*]pyrimidines. Consequently, condensation of the aminoimino compound **18** with either formic acid or methyl formate in benzene at reflux yielded 14-(4-methoxyphenyl)-12-methoxy-14*H*-benzo[*h*]chromeno[3,2-*e*][1,2,4]triazolo-[1,5-*c*]pyrimidine (**19a**), while acylation of **18** with acetyl chloride or acetic anhydride gave the pentacyclic 2-methyltriazolopyrimidine derivative **19b**. Moreover, condensation of **18** with diethyl oxalate and ethyl cyanoacetate afforded the desired products, 2-ethoxycarbonyltriazolopyrimidine **19c** and 2-cyanomethyltriazolopyrimidine derivatives **19d**, respectively. Finally, aroylation of **18** with benzoyl chloride in refluxing dry benzene proceeded readily to give the 2-phenyltriazolopyrimidine derivative **19e**. These results are depicted in Scheme 6.

**Scheme 7:**
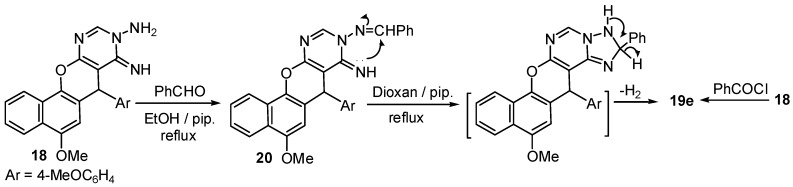
Synthesis of preparation of compounds **19e** and **20**.

Condensation of the aminoimino compound **18** with benzaldehyde in ethanolic piperidine solution under reflux gave the open chain molecule 9-benzylideneamino-8-imino-7-(4-methox-phenyl)-5-methoxy-7*H*-benzo[*h*]chromeno[2,3-*d*]pyrimidine (**20**) [42], while cyclization of **20** in 1,4-dioxane-piperdine solution under reflux gave the cycloaddition product **19e** [42] (Scheme 7), which can also be obtained as described in Scheme 6 from the aroylation of **18** with benzoyl chloride.

**Scheme 8:**
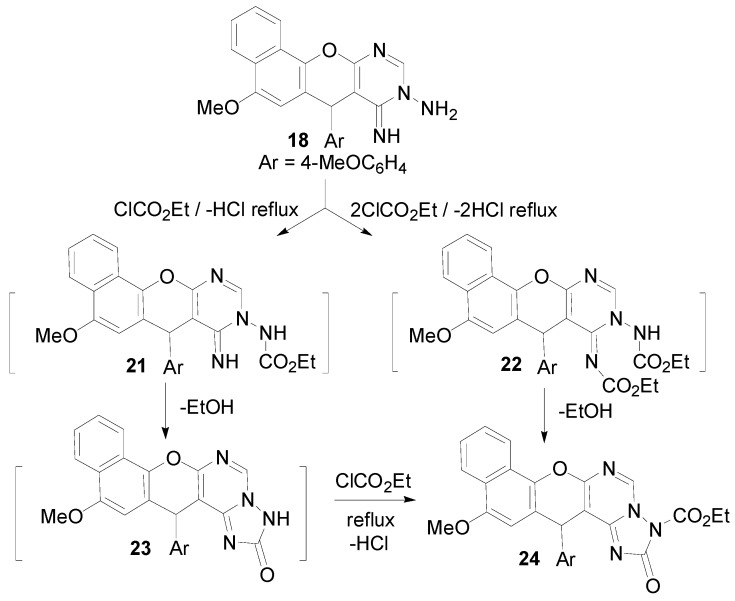
Synthesis of 3-ethoxycarbonyl-14-(4-methoxyphenyl)-12-methoxy-14*H*-benzo[*h*]chromeno-[3,2-*e*][1,2,4]triazolo[1,5-*c*]pyrimidine-2-one (**24**).

Interaction of the aminoimino compound **18** with ethyl chloroformate in dry benzene at reflux afforded a 1:2 adduct, the 3-ethoxycarbonyltriazolopyrimidine-2-one **24** instead of the 1:1 adduct, the triazolopyrimidine-2-one **23** (Scheme 8). The formation of **24** is assumed to proceed via interaction of **18** with one mole of ethyl chloroformate to eliminate HCl and yield the intermediate **21**, which then cyclized to the non-isolable compound **23** via elimination of EtOH. Further interaction of **23** with another mole of ethyl chloroformate eliminated HCl and produced compound **24**. Alternatively, interaction of **18** with two moles of ethyl chloroformate eliminated two molecules of HCl and afforded the intermediate bis-(ethoxycarbonyl) derivative **22**, which then cyclized to **24** with elimination of ethanol, Scheme 8. It is also important to mention that the 4-position of compounds **4**, **6**–**11**, **13**, **14**, the 7-position of compounds **15**–**18**, **20** and 14-position of compounds **19a**–**e**, **24** are chiral centers. The structures of the synthesized compounds were established on the basis of spectral data, IR, ^1^H-NMR, ^13^C-NMR and MS data (see the Experimental Section and Supplementary Materials).

### 2.2. Antitumor Evaluation

**Figure 3:**
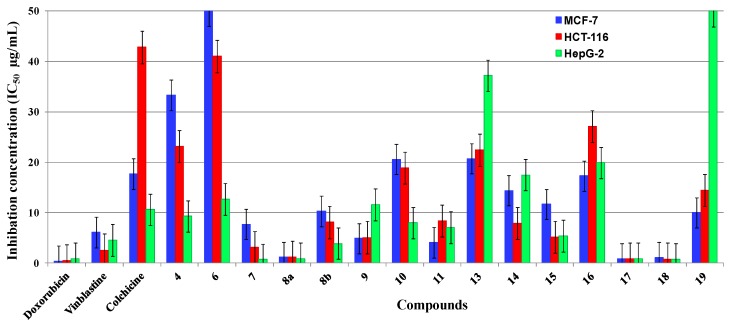
IC_50_ values expressed in (µg/mL) of 3-amino-1*H*-benzo[*f*]chromene derivatives **4a**–**h** and **6a**–**h** against MCF-7, HCT and HepG-2 tumor cells.

Based on the reported antitumor activities of a great number of bioactive compounds incorporating chromene or benzochromene moieties [5,32,33,34,35,36,37,38,39], the target compounds **4**, **6**–**11**, **13**–**18** and **20** were selected to carry out a preliminary screening for their cytotoxic effects against three metastatic human cancer cell lines, including MCF-7 (breast cancer), HCT-116 (human colon cancer) and HepG-2 (liver cancer). The selection of such cell lines and standard drugs was inspired by the declared anticancer activity of a number of chromene and benzochromene derivatives [5,33,34,35,36,37,38,39,40]. The cytotoxic activity was evaluated using the 3-(4,5-dimethylthiazol-2-yl)-2,5-diphenyltetrazolium bromide (MTT) colorimetric assay [43,44]. In vitro cytotoxicity evaluation was performed at the Al-Azhar University Regional Centre for Mycology and Biotechnology (RCMP), under different concentrations (50, 25, 12.5, 6.25, 3.125, 1.56 and 0 µg/mL). Vinblastine, colchicine and doxorubicin are used as reference compounds. The results were expressed as growth inhibitory concentration (IC_50_) values which represent the compound concentrations required to produce a 50% inhibition of cell growth after 24 h of incubation compared to untreated controls as shown in Table 1 and Figure 3.

From the obtained results, it was elucidated that most of the synthesized compounds displayed excellent to modest growth inhibitory activity against the tested cancer cell lines. The investigations indicated that HepG-2 was the cell line most sensitive to the influence of the new derivatives (Table 1). Compounds **17**, **18**, **8a**, **11** and **9** were found to be the most potent derivatives against MCF-7 cancer cells, as they were 6.8, 5.6, 5.1, 1.5, 1.3 and 19.7, 16, 1, 14.8, 4.3, 3.6 times more active than vinblastine and colchicine, respectively (Table 1), while compounds **7**, **20**, **8b**, **15**, **14** and **16** displayed good activity against the MCF-7 cancer cell as they were 2.3, 1.8, 1.7, 1.5, 1.3 and 1.0 times more active than colchicine. Besides, compounds **18**, **17** and **8a** were more potent and efficacious against HCT-116 cancer cells, as they were 3.3, 2.9 and 2.2 times more active than vinblastine and colchicine, respectively. In the meantime, compounds **7**, **9**, **15**, **14**, **8b**, **11**, **20**, **10**, **13**, **4**, **16** and **6** displayed good activity against HCT-116 cancer cells (13.8, 8.4, 8.2, 5.4, 5.3, 5.1, 3.0, 2.3, 1.9, 1.8, 1.6 and 1.1 times more active than colchicine). On the other hand, the cytotoxicity evaluation in the HepG-2 cell line revealed that compounds **7**, **18**, **17** and **8a**,**b** were the most active (6.6, 5.8, 5.1, 5.1, 1.2 and 15.1, 13.3, 11.8, 11.8, 2.7 times more active than vinblastine and colchicine, respectively). Additionally, compounds **15**, **11**, **10** and **4** displayed good activity against the HepG-2 cancer cell, being 2.0, 1.5, 1.3 and 1.1 times more active than colchicine. In the meantime, compound **17** was almost equipotent to doxorubicin against MCF-7 cancer cells, while compound **18** and **17** was almost equipotent to doxorubicin against HCT-116 cancer cells, and finally compounds **7** and **18** displayed significantly good growth inhibitory activity against HepG-2 cancer cells, as they were 1.3 and 1.1 times more active than doxorubicin, while compounds **17** and **8a** were equipotent as doxorubicin.

### 2.3. SAR Studies

The corresponding partition coefficients (log *P*), which are known as an index of lipophilicity, were calculated using ACD/Labs log *P* ver. 14.02, are listed in Table 1. The preliminary structure–activity relationships (SAR) study focused on the effects of the replacement at 2,3-positions or 2,3-positions of the fused rings on the antitumor activities of the synthesized compounds. The study includes a comparison of the cytotoxic activities of compounds **4**, **6** and their analogues against the MCF-7, HCT-116 and HepG-2 cell lines. For instance, the SAR study of compound **4** and its analogues has confirmed that the more potent and efficacious activity than vinblastine and colchicine of compounds **8a**, **11** and **9** against the MCF-7 cancer cells and the good activity of compounds **7** and **8b** against MCF-7 cancer cells as compared to compound **10** and colchicine was attributable mainly to the presence of the -NHAc, -N=CHNH_2_, -N=CHOEt, -N=CHPh and -NAc_2_ moieties at the 2-position and some hydrophobic groups are preferred over others at this 2-position, as indicated by the increasing values of Log *P* shown in Table 1. Besides, blocking the 2-(-NH_2_) group of compound **4** with other hydrophobic moieties, for example with a 2-(-N=CHNMe_2_) as in compound **10**, resulted in the reduction of the potency. Replacement of the 3-cyano with a 3-ester (a hydrophobic group) caused a reduction of the potency of compound **6** as compared to compound **4**, while blocking the 2-(-NH_2_) group of compound **6** with hydrophilic or hydrophobic groups such as (-NHCHO or N=CHNMe_2_) reduced the potency of compound **13** and improved the potency of compound **14** against MCF-7 cancer cells as compared to colchicine. Incorporating a pyrimidine ring at the 2,3-postions of compound **4** with hydrophobic groups (=NH-8, -Me-9) for compound **17** and (=NH-8, -NH_2_-9) for compound **18** resulted in a strong improvement of potency against MCF-7 cancer cells as compared to vinblastine and colchicine, while the presence of a hydrophilic 8-(-C=O) group for compound **15** or a hydrophobic group 8-(-NH_2_) for compound **16** barely reduces their potency while more reduction of potency is observed with hydrophobic groups (=NH-8, -N=CHPh-9) for compound **20** against the MCF-7 cancer cell as compared to colchicine. This behavior suggests that the antitumor activity is significantly affected by the lipophilicity as indicated by a decreasing value of log *P* as shown in Table 1 and hydrophobic groups are more beneficial than hydrophilic groups, as well as the 7*H*-benzo[*h*]-chromeno[2,3-*d*]pyrimidine nucleus is more valuable than the 4*H*-benzo[*h*]chromene nucleus.

Further investigation of the impact of the substitution pattern at the previous positions of the synthesized compounds on the antitumor activities was then conducted. Compound **8a**, bearing a hydrophobic substituent (-NHAc) at the 2-position, exhibited an increase in the activity against HCT-116 cancer cells compared to vinblastine and colchicine, whereas compounds **7**, **9**, **8b**, **11**, **10** and **4** with hydrophobic substituents (-N=CHPh, -N=CHOEt, -NAc_2_, -N=CHNH_2_, -N=CHNMe_2_ and NH_2_) at the 2-position displayed a remarkable enhancement in the antitumor activity against HCT-116 cancer cells compared to colchicine. This behavior suggests that the substitution at 2-position could be tolerated and the incorporation of hydrophobic substituents is beneficial for increasing the value of Log *P* as shown in Table 1. Replacing the 3-cyano- with 3-ester (a hydrophobic group) resulted in a loss of the activity for compound **6** compared to compound **4**. The blocking of the 2-(-NH_2_) group in compound **6** with hydrophilic or hydrophobic groups such as 2(-NHCHO) or 2-(-N=CHNMe_2_) caused a remarkable enhancement in the antitumor activity for compounds **13** and **14** against HCT-116 cancer cells compared to colchicine. The introduction of a pyrimidine ring at the 2,3-postions of compound **4** with a hydrophobic group 8-(=NH), 9-(-NH_2_) for compound **18** and 8-(=NH), 9-(-Me) for compound **17** resulted in a remarkable enhancement of potency against HCT-116 cancer cells as compared to vinblastine and colchicine. In contrast, the presence of a hydrophilic 8-(-C=O) group in compound **15** resulted in a partial loss of the activity. The same behavior has been observed after incorporation of hydrophobic groups (-NH_2_-8; =NH-8, -Me-9; =NH-8, -N=CHPh-9) into compounds **16**, **17** and **20**, hinting that grafting a pyrimidine ring at the 2,3-postions with a lipophilic substituent (hydrophobic group) like imino/amino or imino/methyl groups is more beneficial than other lipophilic substituenta (hydrophilic or hydrophobic groups) like carbonyl, imino or benzylideneamino moieties for the activity by increasing the value of Log *P* as shown in Table 1.

Concerning the activity against HepG-2, compounds **7**, **18**, **17** and **8a**,**b** were the most active analogs through this study with IC_50_ values of 0.7 ± 0.11, 0.8 ± 0.08, 0.9 ± 0.11, 0.9 ± 0.1 and 3.9 ± 0.3, respectively, in comparison to the reference drugs vinblastine and colchicine (IC_50_ = 4.6 ± 0.01 and 10.6 ± 0.04 µg/mL). Additionally, compounds **15**, **11**, **10** and **4** displayed good activity against HepG-2 in comparison to colchicine and the other compounds **9**, **6**, **14**, **16**, **13** and **20**. These results imply that the introduction of a hydrophobic group (-N=CHPh or -NHAc) at the 2-position of the chromene nucleus and the incorporation of a pyrimidine nucleus at the 2,3-positions with a hydrophobic group (=NH-8, -NH_2_-9 or =NH-8, -Me-9) were indispensable for the activities against HepG-2 by decreasing the value of log *P* as shown in Table 1. In addition, compound **17** was found to be the most potent derivative against MCF-7 as compared to doxorubicin, as it was almost equipotent as doxorubicin, while compounds **18** and **17** were almost equipotent as doxorubicin against HCT-116. Besides, compounds **7** and **18** with IC_50_ = 0.7 ± 0.11 and 0.8 ± 0.08 µg/mL displayed significant growth inhibitory activity against HepG-2 in comparison to doxorubicin, and compounds **17** and **8a** were equipotent as doxorubicin, indicating that hydrophobic groups like benzylideneamino and acetylamino moieties at 2-postion is preferred for antitumor activity more than other hydrophobic groups with decreasing value of log *P* as shown in Table 1 and the 4*H*-benzo[*h*]chromene nucleus more significantly than the 7*H*-benzo[*h*]chromeno[2,3-*d*]pyrimidine nucleus. The other compounds showed moderate to fair cytotoxic activities as compared to doxorubicin.

Finally, we can deduce that the substitution pattern at the 2,3-positions or fused rings at the 2,3-positions on the synthesized 4*H*-chromene and pyrimidine moieties are crucial elements for the antitumor activity. The incorporation of pyrimidine rings at the 2,3-postions with groups (=NH-8, -NH_2_-9 and =NH-8, -Me-9) or (-N=CHPh and -NHAc) at the 2-postion of the chromene nucleus is favorable and greatly enriches the activity more than the other hydrophobic and hydrophilic groups tested.

## 3. Experimental Section

### 3.1. General Information

Commercial-grade solvents and reagents were purchased from Sigma-Aldrich (St. Louis, MO, USA) and used without further purification. Melting points were measured with a Stuart Scientific (Stone, Staffordshire, UK) apparatus and are uncorrected. IR spectra were determined as KBr pellets on a FT/IR 460 plus spectrophotometer (Jasco, Tokoyo, Japan). ^1^H-NMR (500 MHz) and ^13^C-NMR spectra (125 MHz) were recorded using an AV 500 MHz spectrometer (Bruker, Billerica, MA, USA). Chemical shifts (*δ*) are expressed in parts per million (ppm). The ^1^H-NMR and ^13^C-NMR spectra of the compounds are provided in the Supplementary Material. The MS were measured using a GC/MS-QP5050A spectrometer (Shimadzu, Tokoyo, Japan). The microwave synthesis was performed using a mono-mode Milestone Sr1 device (Milestone, Shelton, CT, USA) while mass spectra were determined on a Shimadzu GC/MS-QP5050A spectrometer. Elemental analyses were carried out at the Regional Centre for Mycology and Biotechnology (RCMP) at Al-Azhar University (Cairo, Egypt) and the results were within ±0.25% of the theoretical values. Analytical thin layer chromatography (TLC) on silica gel precoated F_254_ (Merck, Billerica, MA, USA) plates was used to check the purity of the compounds.

### 3.2. Synthesis

*2-Amino-4-(4-methoxyphenyl)-6-methoxy-4H-benzo[h]chromene-3-carbonitrile* (**4**). A reaction mixture of 4-methoxy-1-naphthol (**1**, 1.74 g, 0.01 mol), 4-methoxybenzaldehyde (**2**, 1.36 g, 0.01 mol), malononitrile (**3**, 0.66 g, 0.01 mol) and piperidine (0.5 mL) in absolute ethanol (30 mL) was heated under microwave irradiation conditions for 2 min. at 140 °C. After the completion of the reaction, the mixture was cooled at room temperature and the precipitated solid was filtered off, washed with methanol and recrystallized from ethanol to give the desired compound **4** as a colorless solid, yield: 87%, m.p. 180–181 °C; IR (KBr, cm*^−^*^1^): 3443, 3332, 3207 (NH_2_), 3079, 3029 (CH-arom.), 2995, 2895 (CH-aliph.), 2193 (CN); ^1^H-NMR (DMSO-*d*_6_, δ, ppm); 3.72 (s, 3H, OCH_3_), 3.81 (s, 3H, OCH_3_), 4.80 (s, 1H, H-4), 7.07 (bs, 2H, NH_2_, canceled by D_2_O), 6.52–8.11 (m, 9H, Ar-H); ^13^C-NMR (DMSO-*d*_6_, δ, ppm); 40.70 (C-4), 54.96 (CH_3_), 55.64 (CH_3_), 56.43 (C-3), 103.44 (C-5), 114.00 (Ar-C), 118.09 (CN), 121.61 (C-4a), 123.66 (C-10), 124.33 (C-7), 126.10 (C-8), 127.17 (C-6a), 128.30 (C-9), 128.56 (C-10a), 128.77 (Ar-C), 136.74 (Ar-C), 137.64 (C-10b), 151.10 (C-6), 158.12 (Ar-C), 160.25 (C-2); its MS (*m*/*z*), 358 (M^+^, 13.92) with a base peak at 251 (100); C_22_H_18_N_2_O_3_ (358.39); calcd. % C: 73.73, % H: 5.06, % N: 7.82; found; % C: 73.79, % H: 5.11, % N: 7.89.

*Ethyl 2-amino-4-(4-methoxyphenyl)-6-methoxy-4H-benzo[h]chromene-3-carboxylate* (**6**). A reaction mixture of 4-methoxy-1-naphthol (**1**, 1.74 g, 0.01 mol), 4-methoxybenzaldehyde (**2**, 1.36 g, 0.01 mol), ethyl cyanoacetate (**5**, 1.13 g, 0.01 mol) and piperidine (0.5 mL) in absolute ethanol (30 mL) was heated under microwave irradiation conditions for 2 min. at 140 °C. After the completion of the reaction, the mixture was cooled at room temperature and the precipitated solid was filtered off, washed with methanol and recrystallized from ethanol to give the desired compound **6** as a colorless solid, yield: 72%, m.p. 159–60 °C; IR (KBr, cm*^−^*^1^): 3414, 3300 (NH_2_), 3014 (CH-arom.), 2997, 2963, 2875 (CH-aliph.), 1682 (CO); ^1^H-NMR (DMSO-*d*_6_, δ, ppm); 1.12 (t, 3H, CH_3_, *J* = 7.1 Hz), 3.77 (s, 3H, OCH_3_), 3.91 (s, 3H, OCH_3_), 4.11 (q, 2H, CH_2_, *J* = 7.1 Hz), 4.99 (s, 1H, H-4), 7.76 (bs, 2H, NH_2_), 6.45–8.30 (m, 9H, Ar-H); ^13^C-NMR (DMSO-*d*_6_, δ, ppm); 14.31 (CH_3_), 40.11 (C-4), 55.43 (CH_3_), 55.63 (CH_3_), 58.60 (CH_2_), 99.78 (C-3), 103.36 (C-5), 114.89 (Ar-C), 115.18 (CN), 121.98 (C-4a), 122.61 (C-10), 122.97 (C-7), 124.78 (C-8), 126.59 (C-6a), 127.81, (C-9), 128.52 (C-10a), 130.54 (Ar-C), 142.17 (C-10b), 142.61(Ar-C), 148.69 (C-6), 151.75 (Ar-C), 161.60 (C-2); its MS (*m*/*z*), 405 (M^+^, 29.33) with a base peak at 299 (100); C_24_H_23_NO_5_ (405.44); calcd. % C: 71.10, % H: 5.72, % N: 3.45; found; % C: 71.15, % H: 5.73, % N: 3.49.

*2-Benzylideneamino-4-(4-methoxyphenyl)-6-methoxy-4H-benzo[h]chromene-3-carbonitrile* (**7**). A mixture of compound **4** (3.58 g, 0.01 mol), benzaldehyde (1.06 g, 0.01 mol) and piperidine (0.5 mL) was refluxed in ethanol (20 mL) for 2 h. (TLC monitoring). The formed precipitate was filtered, washed with cooled methanol, dried and recrystallized from ethanol to afford **7** as a yellow solid, yield: 75%, m.p. 203–204 °C; IR (KBr, cm*^−^*^1^): 3043, 3000 (CH-arom.), 2980, 2896 (CH-aliph.), 2227 (CN); ^1^H-NMR (DMSO-*d*_6_, δ, ppm); 3.78 (s, 3H, OCH_3_), 3.90 (s, 3H, OCH_3_), 4.86 (s, 1H, H-4), 6.45–8.18 (m, 14H, Ar-H), 8.40 (s, 1H, N=CH); ^13^C-NMR (DMSO-*d*_6_, δ, ppm); 40.01 (C-4), 55.33 (CH_3_), 55.59 (CH_3_), 99.70 (C-3), 103.30 (C-5), 114.33 (Ar-C), 115.12 (CN), 121.96 (C-4a), 122.01 (C-10), 122.87 (C-7), 124.73 (C-8), 126.50 (C-6a), 127.01, (C-9), 127.52 (C-10a), 128.25 (Ar-C), 128.30 (Ar-C), 129.21 (Ar-C), 130.50 (Ar-C), 140.15 (C-10b), 144.60 (Ar-C), 146.66 (C-6), 150.70 (Ar-C), 155.73 (N=CH ), 160.62 (C-2); its MS (*m*/*z*), 446 (M^+^, 1.00) with a base peak at 101 (100); C_29_H_22_N_2_O_3_ (446.50); calcd. % C: 78.01, % H: 4.97, % N: 6.27; found; % C: 78.10, % H: 5.00, % N: 6.31.

*2-Acetylamino-4-(4-methoxyphenyl)-6-methoxy-4H-benzo[h]chromene-3-carbonitrile* (**8a**). A solution of compound **4** (3.58 g, 0.01 mol) was refluxed in acetic anhydride (20 mL) for 1/2 h. The solvent was removed under reduced pressure and the resulting solid was collected and washed with cooled methanol, filtered, dried and recrystallized from ethanol to afford **8a** as a yellow solid, yield: 52%, m.p. 150–151 °C; IR (KBr, cm*^−^*^1^): 3272 (NH), 3021 (CH-arom.), 2995, 2945, 2903, 2845 (CH-aliph.), 2217 (CN), 1699 (CO); ^1^H-NMR (DMSO-*d*_6_, δ, ppm); 2.13 (s, 3H, COCH_3_), 3.76 (s, 3H, OCH_3_), 3.84 (s, 3H, OCH_3_), 5.29 (s, 1H, H-4), 6.53–8.14 (m, 9H, Ar-H), 10.70 (s, 1H, NH); ^13^C-NMR (DMSO-*d*_6_, δ, ppm); 24.50 (CH_3_), 41.84 (C-4), 55.07 (CH_3_), 55.89 (CH_3_), 92.18 (C-3), 102.93 (C-5), 114.37 (Ar-C), 115.51 (CN), 116.39 (C-4a), 120.62 (C-10), 121.72 (C-7), 123.53 (C-6a), 124.57 (C-8), 126.76 (C-9), 127.91 (C-10a), 129.41 (Ar-C), 134.44 (Ar-C), 136.94 (C-10b), 151.74 (C-6), 152.48, (Ar-C), 158.79 (C-2), 170.60 (CO); its MS (*m*/*z*), 400 (M^+^, 22.30) with a base peak at 64 (100); C_24_H_20_N_2_O_4_ (400.43); calcd. % C: 71.99, % H: 5.03, % N: 7.00; found; % C: 71.88, % H: 4.90, % N: 6.97.

*2-Diacetylamino-4-(4-methoxyphenyl)-6-methoxy-4H-benzo[h]chromene-3-carbonitrile* (**8b**). Compound **8b** was obtained via refluxing of **4** (3.58 g, 0.01 mol) with acetic anhydride (20 mL) for 6 h. Reaction work up was performed according to the procedure described for **8a**. **8b** was collected as a yellow solid, yield: 50%, m.p. 172–173 °C; IR (KBr, cm*^−^*^1^): 3012 (CH-arom.), 2945, 2912, 2845 (CH-aliph.), 2219 (CN), 1735 (CO); ^1^H-NMR (DMSO-*d*_6_, δ, ppm); 2.47 (s, 6H, 2COCH_3_), 3.75 (s, 3H, OCH_3_), 3.84 (s, 3H, OCH_3_), 5.29 (s, 1H, H-4), 6.53–8.14 (m, 9H, Ar-H); ^13^C-NMR (DMSO-*d*_6_, δ, ppm); 23.06 (CH_3_), 41.84 (C-4), 55.06 (CH_3_), 55.88 (CH_3_), 92.18 (C-3), 102.92 (C-5), 114.37 (Ar-C), 115.51 (CN), 116.93 (C-4a), 120.61 (C-10), 121.72 (C-7), 123.54 (C-8), 124.57 (C-6a), 126.75 (C-9), 127.90 (C-10a), 129.41 (Ar-C), 134.44 (Ar-C), 136.94 (C-10b), 151.74 (C-6), 152.48 (Ar-C), 158.79 (C-2), 170.50 (CO); its MS (*m*/*z*), 442 (M^+^, 12.30) with a base peak at 294 (100); C_26_H_22_N_2_O_5_ (442.15); calcd. % C: 70.58, % H: 5.01, % N: 6.33; found; % C: 70.61, % H: 5.03, % N: 6.36.

*2-Ethoxymethyleneamino-4-(4-methoxyphenyl)-6-methoxy-4H-benzo[h]chromene-3-carbonitrile* (**9**). A mixture of **4** (3.58 g, 0.01 mol) with triethyl orthoformate (1.48 g, 0.01 mol) and acetic anhydride (30 mL) was refluxed for 2 h. The solvent was removed under reduced pressure and the resulting solid was washed with methanol and recrystallized from benzene to give **9** as a yellow solid, yield: 86%, m.p. 145–146 °C; IR (KBr, cm*^−^*^1^): 3012 (CH-arom.), 2962, 2928, 2870, 2836 (CH-aliph.), 2206 (CN); ^1^H-NMR (DMSO-*d*_6_, δ, ppm); 1.36 (t, 3H, CH_3_, *J* = 7.2 Hz), 3.73 (s, 3H, OCH_3_), 3.79 (s, 3H, OCH_3_), 4.39 (q, 2H, CH_2_, *J* = 7.2 Hz), 4.97 (s, 1H, H-4), 6.43–8.13 (m, 9H, Ar-H), 8.89 (s, 1H, N=CH); ^13^C-NMR (DMSO-*d*_6_, δ, ppm); 13.89 (CH_3_), 41.77 (C-4), 55.03 (CH_3_), 55.67 (CH_3_), 63.82 (CH_2_), 80.07 (C-3), 103.12 (C-5), 114.20 (Ar-C), 116.62 (CN), 118.16 (C-4a), 121.55 (C-10), 123.87 (C-7), 124.55 (C-8), 126.38 (C-6a), 127.45 (C-9), 128.29 (C-10a), 129.14 (Ar-C), 135.93 (Ar-C), 136.86 (C-10b), 151.64 (C-6), 157.08 (Ar-C),158.50 (C-2), 161.45 (N=CH); its MS (*m*/*z*), 414 (M^+^, 88.03) with a base peak at 308 (100); C_25_H_22_N_2_O_4_ (414.45); calcd. % C: 72.45, % H: 5.35, % N: 6.76; found; % C: 72.43, % H: 5.33, % N: 6.73.

*2-Dimethylaminomethyleneamino-4-(4-methoxyphenyl)-6-methoxy-4H-benzo[h]chromene-3-carbonitrile* (**10**) **Method (a)**: A mixture of **4** (3.58 g, 0.01 mol) with dimethylformaide-dipentylacetal (DMF-DPA) (2.16 g, 0.01 mol) and benzene (30 mL) was refluxed for 3 h. The solvent was removed under reduced pressure and the resulting solid was recrystallized from benzene to give **10** as a colorless solid, yield: 90%, m.p. 215–216 °C; IR (KBr, cm*^−^*^1^): 3062 (CH-arom.), 2995, 2962, 2934, 2878, 2836 (CH-aliph.), 2189 (CN); ^1^H-NMR (DMSO-*d*_6_, δ, ppm); 3.32, 3.34 (s, 6H, 2CH_3_), 3.73 (s, 3H, OCH_3_), 3.81 (s, 3H, OCH_3_), 4.90 (s, 1H, H-4), 6.50–8.39 (m, 9H, Ar-H), 8.62 (s, 1H, N=CH); ^13^C-NMR (DMSO-*d*_6_, δ, ppm); 34.25 (CH_3_), 42.01 (C-4), 55.01 (CH_3_), 55.67 (CH_3_), 72.45 (C-3), 103.46 (C-5), 114.07 (Ar-C), 117.40 (CN), 120.35 (C-4a), 121.27 (C-10), 121.39 (C-7), 124.03 (C-8), 124.44 (C-6a), 126.17 (C-9), 127.25 (C-10a), 128.79 (Ar-C), 137.12 (Ar-C), 137.21 (C-10b), 151.14 (C-6), 154.41 (Ar-C), 158.21 (C-2), 159.45 (N=CH); its MS (*m*/*z*), 413 (M^+^, 1.00) with a base peak at 77 (100); C_25_H_23_N_3_O_3_ (413.47); calcd. % C: 72.62, % H: 5.61, % N: 10.16; found; % C: 72.58, % H: 5.58, % N: 10.20. **Method (b)**: A mixture of imadate **9** (4.14 g, 0.01 mol) and dimethylamine (0.45 g, 0.01 mol) in methanol (30 mL), was stirred at room temperature for 1 h then left overnight to precipitate. The solid product was collected by filtration, washed with methanol and recrystallized from proper solvent to afford **10**.

*2-Aminomethyleneamino-4-(4-methoxyphenyl)-6-methoxy-4H-benzo[h]chromene-3-carbonitrile* (**11**). A mixture of imidate **9** (4.14 g, 0.01 mol) and NH_3_ gas bubbled in methanol (30 mL) was stirred at room temperature for 1 h. (TLC monitoring) and the mixture was left overnight. The solid product was collected by filtration, washed with methanol and recrystallized from dioxane to give **11** as a colourless solid, yield: 81%, m.p. 245–246 °C; IR (KBr, cm*^−^*^1^): 3468, 3309, 3165 (NH_2_), 3004 (CH-arom.), 2962, 2920, 2828 (CH-aliph.), 2203 (CN); its MS (*m*/*z*), 385 (M^+^, 3.26) with a base peak at 279 (100); C_23_H_19_N_3_O_3_ (385.42); calcd. % C: 71.67, % H: 4.97, % N: 10.90; found; % C: 71.59, % H: 4.94, % N: 10.85.

*Ethyl 4-(4–4-methoxphenyl)-2-formamido-6-methoxy-4H-benzo[h]chromene-3-carboxylate* (**13**). Compound **13** was prepared from **6** (4.05 g, 0.01 mol), triethyl orthoformate (1.48 g, 0.01 mol) and acetic anhydride (30 mL) according to the procedure described for compound **9**. Compound **13** was recrystallized from benzene as a pale yellow solid, yield: 70%, m.p. 150–151 °C; IR (KBr, cm*^−^*^1^): 3405 (NH), 3071 (CH-arom.), 2937, 2825 (CH-aliph.), 1682 (CO), 1709 (CO); ^1^H-NMR (DMSO-*d*_6_, δ, ppm); 1.18 (t, 3H, CH_3_, *J* = 7.2 Hz), 3.69 (s, 3H, OCH_3_), 3.88 (s, 3H, OCH_3_), 4.11 (q, 2H, CH_2_, *J* = 7.2 Hz), 5.11 (s, 1H, H-4), 6.73–8.28 (m, 9H, Ar-H), 10.59 (s, 1H, CHO), 10.70 (bs, 1H, NH); ^13^C-NMR (DMSO-*d*_6_, δ, ppm); 13.99 (CH_3_), 40.33 (C-4), 54.95 (CH_3_), 55.84 (CH_3_), 60.40 (CH_2_), 92.14 (C-3), 103.55 (C-5), 113.55 (Ar-C), 120.32 (C-4a), 121.57 (C-10), 124.50 (C-7), 126.19 (C-8), 127.57 (C-6a), 128.18 (C-9), 128.39 (C-10a), 128.55 (Ar-C), 137.96 (C-10b), 139.70 (Ar-C), 151.97 (C-6), 152.28 (C-2), 157.55 (Ar-C), 166.45 (CO), 167.50 (CO); its MS (*m*/*z*), 433 (M^+^, 1.15) with a base peak at 68 (100); C_25_H_23_NO_6_ (433.45); calcd. % C: 69.27, % H: 5.35, % N: 3.23; found; % C: 69.19, % H: 5.39, % N: 3.18.

*Ethyl 2-dimethylaminomethyleneamino-4-(4-methoxyphenyl)-6-methoxy-4H-benzo[h]chromene-3-carboxylate* (**14**). Compound **14** was prepared from **6** (4.05 g, 0.01 mol), dimethylformamide-dipentylacetal (DMF-DPA) (2.16 g, 0.01 mol) and benzene (30 mL) according to the procedure described for compound **10** (**Method (a)**). Compound **14** was recrystallized from benzene as a pale yellow solid, yield: 68%, m.p. 120–121 °C; IR (KBr, cm*^−^*^1^): 3062, 3029 (CH-arom.), 2932, 2878, 2828 (CH-aliph.), 1660 (CO); ^1^H-NMR (DMSO-*d*_6_, δ, ppm); 1.16 (t, 3H, CH_3_, *J* = 7.2 Hz), 3.34, 3.17 (s, 6H, 2CH_3_), 3.67 (s, 3H, OCH_3_), 3.87 (s, 3H, OCH_3_), 4.00 (q, 2H, CH_2_, *J* = 7.2 Hz), 5.11 (s, 1H, H-4), 6.76–8.25 (m, 9H, Ar-H), 8.22 (s, 1H, N=CH); ^13^C-NMR (DMSO-*d*_6_, δ, ppm); 14.14 (CH_3_), 34.23 (CH_3_), 42.12 (C-4), 54.89 (CH_3_), 55.68 (CH_3_), 58.82 (CH_2_), 90.00 (C-3), 103.76 (C-5), 113.58 (Ar-C), 120.81 (C-4a), 121.53 (C-10), 123.96 (C-7), 124.04 (C-8), 125.54 (C-6a), 126.92 (C-9), 128.27 (C-10a), 128.33 (Ar-C), 137.89 (C-10b), 139.32 (Ar-C), 150.97 (C-6), 155.72 (Ar-C), 157.58 (C-2), 160.73 (N=CH), 167.11 (CO); its MS (*m*/*z*), 460 (M^+^, 52.99) with a base peak at 336 (100); C_27_H_28_N_2_O_5_ (460.52); calcd. % C: 70.42, % H: 6.13, % N: 6.08; found; % C: 70.37, % H: 6.08, % N: 6.03.

*7-(4-Methoxyphenyl)-5-methoxy-7H,9H-benzo[h]chromeno[2,3-d]pyrimidin-8-one* (**15**). A mixture of compound **4** (3.58 g, 0.01 mol) and formic acid (4.6 g, 1 mol) was refluxed for 3–5 h. (TLC monitoring). The excess of formic acid was removed under reduced pressure and the resulting solid was recrystallized from ethanol/dioxan to give **15** as a yellow solid, yield: 87%, m.p. 170–171 °C; IR (KBr, cm*^−^*^1^): 3501 (NH), 3077, 3001 (CH-arom.), 2958, 2932, 2891 (CH-aliph.), 1763 (CO); its MS (*m*/*z*), 386 (M^+^, 1.00) with a base peak at 75; C_23_H_18_N_2_O_4_ (386.40); calcd. % C: 71.49, % H: 4.70, % N: 7.25; found; % C: 71.52, % H: 4.81, % N: 7.34.

*8-Amino-7-(4-methoxyphenyl)-5-methoxy-7H-benzo[h]chromeno[2,3-d]pyrimidine* (**16**). Compound **11** (3.85 g, 0.01 mol) was refluxed in ethanol in the presence of pipridine (0.5 mL) for 2 h. The formed precipitate was collected by filtration, washed with methanol and recrystallized from ethanol to give **16** as a colorless solid, yield: 80%, m.p. 277–278 °C; IR (KBr, cm*^−^*^1^): 3468, 3414, 3305 (NH_2_), 3097 (CH-arom.), 2995, 2970, 2928, 2836 (CH-aliph.); ^1^H-NMR (DMSO-*d*_6_, δ, ppm); 3.68 (s, 3H, OCH_3_), 3.89 (s, 3H, OCH_3_), 5.29 (s, 1H, H-7), 7.29 (s, 2H, NH_2_), 8.26 (s, 1H, H-10), 6.59–8.25 (m, 9H, Ar-H); ^13^C-NMR (DMSO-*d*_6_, δ, ppm); 38.00 (C-7), 54.97 (CH_3_), 55.74 (CH_3_), 95.86 (C-7a), 103.36 (C-6), 114.00 (Ar-C), 119.47 (C-6a), 120.68 (C-1), 121.64 (C-4), 124.20 (C-3), 124.38 (C-4a), 126.17 (C-2), 127.40 (C-1a), 128.63 (Ar-C), 136.16 (C-1b), 137.63 (Ar-C), 151.26 (C-5), 156.39 (C-10), 158.10 (Ar-C), 162.23 (C-11a), 162.63 (C-8); its MS (*m*/*z*), 385 (M^+^, 78.40) with a base peak at 278 (100); C_23_H_19_N_3_O_3_ (385.42); calcd. % C: 71.67, % H: 4.97, % N: 10.90; found; % C: 71.58, % H: 4.89, % N: 10.81.

*7-(4-Methoxyphenyl)-5-methoxy-8-imino-9-methyl-7H-benzo[h]chromeno[2,3-d]pyrimidine* (**17**). This compound was prepared from the imidate **9** (4.14 g, 0.01 mol), methylamine (0.31 g, .01 mol) and methanol (30 mL) according to the procedure described for compound **11**. Compound **17** was recrystallized from dioxane as a colorless solid, yield: 79%, m.p. 199–200 °C; IR (KBr, cm*^−^*^1^): 3331 (NH), 3062 (CH-arom.), 2995, 2965, 2924, 2836 (CH-aliph.); its MS (*m*/*z*), 399 (M^+^, 92.55) with a base peak at 76 (100); C_24_H_21_N_3_O_3_ (399.44); calcd. % C: 72.16, % H: 5.30, % N: 10.52; found; % C: 72.21, % H: 5.36, % N: 10.58.

*9-Amino-7-(4-methoxyphenyl)-5-methoxy-8-imino-7H-benzo[h]chromeno[2,3-d]pyrimidine* (**18**). Prepared from the imidate **9** (4.14 g, 0.01 mol), hydrazine hydrate (0.5 g, 0.01 mol) and methanol (30 mL) according to the procedure described for compound **11**. Compound **18** was recrystallized from benzene as a colorless solid, yield: 88%, m.p. 210–211 °C; IR (KBr, cm*^−^*^1^): 3347, 3301, 3263 (NH and NH_2_), 2994, 2965, 2932, 2838 (CH-aliph.); ^1^H-NMR (DMSO-*d*_6_, δ, ppm); 3.68 (s, 3H, OCH_3_), 3.86 (s, 3H, OCH_3_), 5.23 (s, 1H, H-7), 5.70 (bs, 2H, NH_2_), 6.84 (bs, 1H, NH), 7.58 (s, 1H, H-10), 8.21–6.60 (m, 9H, Ar-H); ^13^C-NMR (DMSO-*d*_6_, δ, ppm); 40.04 (C-7), 55.97 (CH_3_), 55.69 (CH_3_), 98.50 (C-7a), 103.75 (C-6), 114.12 (Ar-C), 118.50 (C-6a), 121.62 (C-1), 124.02 (C-4), 124.30 (C-3), 126.09 (C-4a), 127.31 (C-2), 128.29 (C-1a), 128.83 (Ar-C), 136.38 (C-1b), 137.18 (Ar-C), 149.10 (C-10), 151.29 (C-5), 155.30 (C-11a), 157.50 (Ar-C), 159.00 (C-8); its MS (*m*/*z*), 400 (M^+^, 11.16) with a base peak at 76 (100); C_23_H_20_N_4_O_3_ (400.43); calcd. % C: 68.99, % H: 5.03, % N: 13.99; found; % C: 68.91, % H: 4.99, % N: 13.89.

*14-(4-Methoxyphenyl)-12-methoxy-14H-benzo[h]chromeno[3,2-e][1,2,4]triazolo[1,5-c]pyrimidine* (**19a**). A mixture of the aminoimino compound **18** (4.00 g, 0.01 mol) and formic acid or methyl formate (0.46 g, or 0.6 g 0.01 mol) in dry benzene (30 mL) was refluxed for 5 h (TLC monitoring). The solvent was removed under reduced pressure and the resulting solid was recrystallized from dioxane to give (**19a**) as a colorless solid, yield: 88%, m.p. 225–226 °C; IR (KBr, cm*^−^*^1^): 3006 (CH-arom.), 2975, 2932, 2837 (CH-aliph.), 1653 (C=N); its MS (*m*/*z*), 410 (M^+^, 1) with a base peak at 76 (100); C_24_H_18_N_4_O_3_ (410.42); calcd. % C: 70.23, % H: 4.42, % N: 13.65; found; % C: 70.17, % H: 4.38, % N: 13.61.

*14-(4-Methoxyphenyl)-12-methoxy-2-methyl-14H-benzo[h]chromeno[3,2-e][1,2,4]triazolo[1,5-c]pyrimidine* (**19b**). A mixture of the aminoimino compound **18** (4.00 g, 0.01 mol) and acetyl chloride or acetic anhydride (30 mL) was refluxed for 2 h (TLC monitoring). The solvent was removed under reduced pressure and the resulting solid was recrystallized from dioxane to give **19b** as a colorless solid, yield: 95%, m.p. 225–226 °C; IR (KBr, cm*^−^*^1^): 3010 (CH-arom.), 2970, 2912, 2837 (CH-aliph.), 1622 (C=N); its MS (*m*/*z*), 424 (M^+^, 56.47) with a base peak at 318 (100); C_25_H_20_N_4_O_3_ (424.45); calcd. % C: 70.74, % H: 4.75, % N: 13.20; found; % C: 70.80, % H: 4.81, % N: 13.27.

*2-Ethoxycarbony-14-(4-methoxyphenyl)-12-methoxy-14H-benzo[h]chromeno[3,2-e][1,2,4]triazolo[1,5-c]pyri-midine* (**19c**). A mixture of the aminoimino compound **18** (4.00 g, 0.01 mol) and diethyl oxalate (1.46 g, 0.01 mol) in ethanol (30 mL) was refluxed for 2 h (TLC monitoring). The solvent was removed under reduced pressure and the resulting solid was recrystallized from ethanol/benzene to give **19c** as a colorless solid, yield: 79%, m.p. 225–226 °C; IR (KBr, cm*^−^*^1^): 3071 (CH-arom.), 2970, 2937, 2836 (CH-aliph.), 1745 (CO), 1623 (C=N); ^1^H-NMR (DMSO-*d*_6_, δ, ppm); 1.36 (t, 3H, CH_3_, *J* = 7.2 Hz), 3.66 (s, 3H, OCH_3_), 3.92 (s, 3H, OCH_3_), 4.42 (q, 2H, CH_2_, *J* = 7.2 Hz), 5.87 (s, 1H, H-14), 6.80–8.34 (m, 9H, Ar-H), 9.80 (s, 1H, H-5),; ^13^C-NMR (DMSO-*d*_6_, δ, ppm); 13.98 (CH_3_), 40.04 (C-14), 54.96 (CH_3_), 55.89 (CH_3_), 61.91 (CH_2_), 102.52 (C-13), 103.67 (Ar-C), 114.03 (C-13a), 118.00 (C-14a), 120.58 (C-8), 121.76 (C-11), 124.03 (C-10), 124.54 (C-11a), 126.45 (C-9), 127.68 (C-8a), 128.96 (Ar-C), 136.00 (Ar-C), 137.74 (C-5), 141.09 (C-8b), 151.93 (C-14b), 152.94 (C-12), 154.49 (C-2), 157.42 (Ar-C), 158.18 (C-6a), 159.48 (CO); its MS (*m*/*z*), 482 (M^+^, 1.39) with a base peak at 76 (100); C_27_H_22_N_4_O_5_ (482.49); calcd. % C: 67.21, % H: 4.60, % N: 11.61; found; % C: 67.28, % H: 4.67, % N: 11.67.

*2-Cyanomethyl-14-(4-methoxyphenyl)-12-methoxy-14H-benzo[h]chromeno[3,2-e][1,2,4]triazolo[1,5-c]pyri-midine* (**19d**). Compound **19d** was prepared from the aminoimino compound **18** (4.00 g, 0.01 mol), ethyl cyanoacetate (1.13 g, 0.01 mol) and ethanol (30 mL) according to the procedure described for compound **19c**. Compound **19d** was recrystallized from ethanol/benzene as colorless solid, yield: 85%, m.p. 235–236 °C; IR (KBr, cm*^−^*^1^): 3097, 3000 (CH-arom.), 2975, 2928, 2836 (CH-aliph.), 2258 (CN), 1652 (C=N); ^1^H-NMR (DMSO-*d*_6_, δ, ppm); 3.45 (s, 2H, CH_2_), 3.68 (s, 3H, OCH_3_), 3.92 (s, 3H, OCH_3_), 5.53 (s, 1H, H-14), 6.60–8.26 (m, 9H, Ar-H), 8.53 (s, 1H, H-5); ^13^C-NMR (DMSO-*d*_6_, δ, ppm); 25.05 (CH_2_), 37.95 (C-14), 55.00 (CH_3_), 55.89 (CH_3_), 97.65 (C-13), 102.78 (C-13a), 114.21 (Ar-C), 117.83 (CN), 118.28 (C-14a), 120.46 (C-8), 121.74 (C-11), 123.88 (C-10), 124.47 (C-11a), 126.52 (C-9), 127.84 (C-8a), 128.71 (Ar-C), 134.55 (Ar-C), 136.86 (C-5), 151.08 (C-8b), 152.21 (C-14b), 153.32 (C-2), 158.48 (C-12), 158.99 (Ar-C), 167.35 (C-6a); its MS (*m*/*z*), 449 (M^+^, 100); C_26_H_19_N_5_O_3_ (449.46); calcd. % C: 69.48, % H: 4.26, % N: 15.58; found; % C: 69.51, % H: 4.29, % N: 15.62.

*14-(4-Methoxyphenyl)-12-methoxy-2-phenyl-14H-benzo[h]chromeno[3,2-e][1,2,4]triazolo[1,5-c]pyrimidine* (**19e**). **Method (a):** Compound **19e** was prepared from the aminoimino compound **18** (4.00 g, 0.01 mol), benzoyl chloride (0.01 mol) according to the procedure described for compound **19a**. Compound **19e** was recrystallized from benzene as a colourless solid, yield: 88%, m.p. 289–290 °C; IR (KBr, cm*^−^*^1^): 3017, 3020 (CH-arom.), 2975, 2918, 2826 (CH-aliph.), 1620 (C=N); ^1^H-NMR (DMSO-*d*_6_, δ, ppm); 3.65 (s, 3H, OCH_3_), 3.91 (s, 3H, OCH_3_), 5.90 (s, 1H, H-14), 6.83–8.36 (m, 14H, Ar-H), 9.69 (s, 1H, H-5); ^13^C-NMR (DMSO-*d*_6_, δ, ppm); 40.03 (C-14), 54.93 (CH_3_), 55.87 (CH_3_), 101.81 (C-13), 103.84 (C-13a), 113.94 (Ar-C), 117.88 (C-14a), 120.63 (C-8), 121.76 (C-11), 124.07 (C-10), 124.59 (C-11a), 126.41 (C-9), 127.16 (C-8a), 127.62 (Ar-C), 129.05 (Ar-C), 129.14 (Ar-C), 129.68 (Ar-C), 136.10 (Ar-C), 138.12 (C-5), 140.06 (C-8b), 151.82 (C-14b), 153.19 (C-12), 153.98 (Ar-C) 158.12 (C-2), 165.47 (C-6a); its MS (*m*/*z*), 486 (M^+^, 40.04) with a base peak at 380 (100); C_30_H_22_N_4_O_3_ (486.52); calcd. % C: 74.06, % H: 4.56, % N: 11.52; found; % C: 74.00, % H: 4.61, % N: 11.58. **Method (b)**: A mixture of compound **20** (4.88 g, 0.01 mol), 1,4-dioxine (20 mL) and piperidine (0.5 mL) was refluxed for 2 h (TLC monitoring). The solvent was removed under reduced pressure and the resulting solid was recrystallized from ethanol/benzene to give (**19e**), yield: 88%.

*9-Benzylideneamino-7-(4-methoxyphenyl)-5-methoxy-8-imino-7H-benzo[h]chromeno[2,3-d]pyrimidine* (**20**). A mixture of aminoimino compound **18** (4.00 g, 0.01 mol) and benzaldehyde (1.06 g, 0.01 mol) in ethanol (30 mL) and piperidine (0.5 mL) was refluxed for 2 h. The solvent was removed under reduced pressure and the resulting solid was recrystallized from dioxane to give the open chain product **20** as a pale yellow solid, yield: 98%, m.p. 218–219 °C; IR (KBr, cm*^−^*^1^): 3209 (NH), 3054, 3009, 3000 (CH-arom.), 2937, 2894 (CH-aliph.), 1649 (C=N); its MS (*m*/*z*), 488 (M^+^, 9.74) with a base peak at 385 (100); C_30_H_24_N_4_O_3_ (488.54); calcd. % C: 73.76, % H: 4.95, % N: 11.47; found; % C: 73.69, % H: 4.89, % N: 11.41.

*3-Ethoxycarbonyl-14-(4-Methoxyphenyl)-12-methoxy-14H-benzo[h]chromeno[3,2-e][1,2,4]triazolo[1,5-c]py-rimidine-2-one* (**24**). A mixture of the aminoimino compound **18** (4.00 g, 0.01 mol) and ethyl chloroformate (0.015 mol) in dry benzene (30 mL) was refluxed for 2 hr (TLC monitoring). The solid product was collected by filtration and recrystallized from benzene to give **24** as a colourless solid, yield: 60%, m.p. >300 °C; IR (KBr, cm*^−^*^1^): 3062 (CH-arom.), 2975, 2962, 2920, 2839 (CH-aliph.), 1763 (CO ester), 1730 (CO); ^1^H-NMR (DMSO-*d*_6_, δ, ppm); 1.34 (t, 3H, CH_3_, *J* = 7.2 Hz), 3.67 (s, 3H, OCH_3_), 3.89 (s, 3H, OCH_3_), 4.44 (q, 2H, CH_2_, *J* = 7.2 Hz), 5.44 (s, 1H, H-14), 6.81–8.28 (m, 9H, Ar-H), 9.33 (s, 1H, H-5); ^13^C-NMR (DMSO-*d*_6_, δ, ppm); 13.98 (CH_3_), 37.78 (C-14), 55.02 (CH_3_), 55.92 (CH_3_), 64.48 (CH_2_), 98.18 (C-14a), 103.96 (C-13), 113.98 (Ar-C), 118.14 (C-13a), 120.50 (C-8), 121.75 (C-11), 123.91 (C-10), 124.46 (C-11a), 126.42 (C-9), 127.80 (C-8a), 129.24 (Ar-C), 137.58 (C-8b), 141.52 (Ar-C), 145.40 (C-5), 148.28 (C-12), 151.99 (C-6a), 152.17 (Ar-C), 154.83 (C-14b), 158.24 (CO), 158.28 (CO); its MS (*m*/*z*), 498 (M^+^, 1) with a base peak at 93 (100); C_27_H_22_N_4_O_6_ (486.52); calcd. % C: 65.05, % H: 4.45, % N: 11.24; found; % C: 65.00, % H: 4.41, % N: 11.21.

### 3.3. Antitumor Activity Assay

#### 3.3.1. Cell Culture

The tumor cell lines breast adenocarcinoma (MCF-7), human colon carcinoma (HCT-116) and hepatocellular carcinoma (HepG-2) were obtained from the American Type Culture Collection (ATCC, Rockville, MD, USA). The cells were grown on RPMI-1640 medium supplemented with 10% inactivated fetal calf serum and 50 µg/mL gentamycin. The cells were maintained at 37 °C in a humidified atmosphere with 5% CO_2_ and were subculture two to three times a week.

#### 3.3.2. Cytotoxicity Evaluation Using Viability Assay

The tumor cell lines were suspended in medium at concentration 5 × 10^4^ cell/well in Corning 96-well tissue culture plates and then incubated for 24 h. The tested compounds with concentrations ranging from 0 to 50 μg/mL were then added into 96-well plates (six replicates) to achieve different conc. for each compound. Six vehicle controls with media or 0.5% DMSO were run for each 96 well plate as a control. After incubating for 24 h, the numbers of viable cells were determined by the MTT test. Briefly, the media was removed from the 96 well plates and replaced with 100 μL of fresh culture RPMI 1640 medium without phenol red then 10 µL of the 12 mM MTT stock solution (5 mg of MTT in 1 mL of PBS) to each well including the untreated controls. The 96-well plates were then incubated at 37 °C and 5% CO_2_ for 4 h. An 85-μL aliquot of the media was removed from the wells, and 50 µL of DMSO was added to each well and mixed thoroughly with the pipette and incubated at 37 °C for 10 min. Then, the optical density was measured at 590 nm with the microplate reader (Sunrise, TECAN, Inc., Morrisville, NC, USA) to determine the number of viable cells and the percentage of viability was calculated as [1 − (ODt/ODc)] × 100% where ODt is the mean optical density of wells treated with the tested sample and ODc is the mean optical density of untreated cells. The relation between surviving cells and drug concentration is plotted to get the survival curve of each tumor cell line after treatment with the specified compound. The 50% inhibitory concentration (IC_50_), the concentration required to cause toxic effects in 50% of intact cells, was estimated from graphic plots of the dose response curve for each conc. using GraphPad Prism software [43,44] (San Diego, CA, USA).

## 4. Conclusions

The synthesis of new compounds with potential applications as drug replacements is an area of high interest in literature in order to overcome the drug resistance issue. For that reason, novel 4*H*-benzo[*h*]chromene-, 7*H*-benzo[*h*]chromeno[2,3-*d*]pyrimidine- and 14*H*-benzo[*h*]-chromeno[3,2-*e*]-[1,2,4]triazolo[1,5-*c*]pyrimidine derivatives have been synthesized, starting from 2-amino-4-(4-methoxyphenyl)-6-methoxy-4*H*-benzo[*h*]chromene-3-carbonitrile and ethyl 2-amino-4-(4-methoxy-phenyl)-6-methoxy-4*H*-benzo[*h*]chromene-3-carboxylate. The new molecules have been evaluated for their antitumor activities against three cancer cell lines: breast adenocarcinoma (MCF-7), human colon carcinoma (HCT-116) and hepatocellular carcinoma (HepG-2). This pharmacological study was also undertaken to evaluate the effects of the substituents and the pyrimidine rings on the antitumor activities. The obtained results have shown that most of these synthesized compounds exhibited good antitumor activities towards the tested cell lines. The SAR study along with the biological assay confirmed that the incorporation of pyrimidine rings at the 2,3-positions with 8-(=NH), 9-(-NH_2_) and 8-(=NH), 9-(-Me) or (-N=CHPh and -NHAc) at the 2-position of the chromene nucleus enhances the activity more than other groups.

## Figures and Tables

**Table 1:**
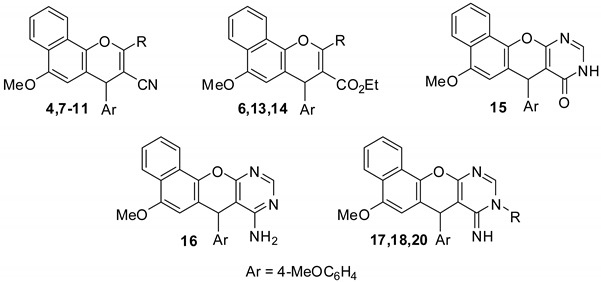
Cytotoxic activity of target compounds via MCF-7, HCT-116 and HepG-2 tumor cells.

Compound	R	IC_50_ (µg/mL) ^a^			log *P*
		MCF-7	HCT-116	HepG-2	
**4**	NH_2_	33.3 ± 0.2	23.2 ± 0.29	9.3 ± 0.4	4.20 ± 0.50
**6**	NH_2_	w	41.0 ± 0.13	12.7 ± 0.15	4.58 ± 0.45
**7**	N=CHPh	7.7 ± 0.14	3.1 ± 0.23	0.7 ± 0.11	6.89 ± 0.63
**8a**	NHAc	1.2 ± 0.35	1.2 ± 0.2	0.9 ± 0.1	4.36 ± 0.50
**8b**	NAc_2_	10.3 ± 0.14	8.1 ± 0.18	3.9 ± 0.3	3.81 ± 0.67
**9**	N=CHOEt	4.9 ± 0.14	5.1 ± 0.36	11.6 ± 0.04	6.12 ± 0.63
**10**	N=CHNMe_2_	20.6 ± 0.01	18.9 ± 0.15	8.0 ± 0.17	4.92 ± 0.64
**11**	N=CHNH_2_	4.1 ± 0.16	8.4 ± 0.02	7.1 ± 0.04	4.62 ± 0.63
**13**	NHCHO	20.7 ± 0.23	22.4 ± 0.18	37.2 ± 0.2	4.57 ± 0.46
**14**	N=CHNMe_2_	14.4 ± 0.01	7.9 ± 0.05	17.5 ± 0.01	5.31 ± 0.63
**15**	-	11.7 ± 0.97	5.2 ± 0.29	5.4 ± 0.07	3.27 ± 0.56
**16**	-	17.3 ± 0.58	27.1 ± 0.13	19.9 ± 0.25	3.72 ± 0.69
**17**	Me	0.9 ± 0.06	0.9 ± 0.05	0.9 ± 0.11	3.52 ± 0.75
**18**	NH_2_	1.1 ± 0.14	0.8 ± 0.12	0.8 ± 0.08	3.00 ± 0.76
**20**	N=CHPh	10.0 ± 0.9	14.5 ± 0.5	w	5.61 ± 0.78
Vinblastine	-	6.1 ± 0.03	2.6 ± 0.08	4.6 ± 0.01	4.58 ± 0.90
Colchicine	-	17.7 ± 0.01	42.8 ± 0.02	10.6 ± 0.04	0.92 ± 0.92
Doxorubicin	-	0.4 ± 0.01	0.5 ± 0.02	0.9 ± 0.04	2.82 ± 1.30

^a^ IC_50_ values expressed in µg/mL as the mean values of triplicate wells from at least three experiments and are reported as the mean ± standard error. w = weak activity (IC_50_ ≥ 75 µg/mL).

## Supplementary Materials

The supplementary materials are available online.
