# Supplementary material for: Design of New Benzo[h]chromene Derivatives: Antitumor Activities and Structure-Activity Relationships of the 2,3-Positions and Fused Rings at the 2,3-Positions
Source: Molecules. 2017 Mar 18;22(3):479. doi: 10.3390/molecules22030479 (PMC6155235; doi:10.3390/molecules22030479)
Supplement: Supplementary file 1 [file molecules-22-00479-s001.zip › molecules-178589-supplementary/13C NMR of compound 19e.pdf]

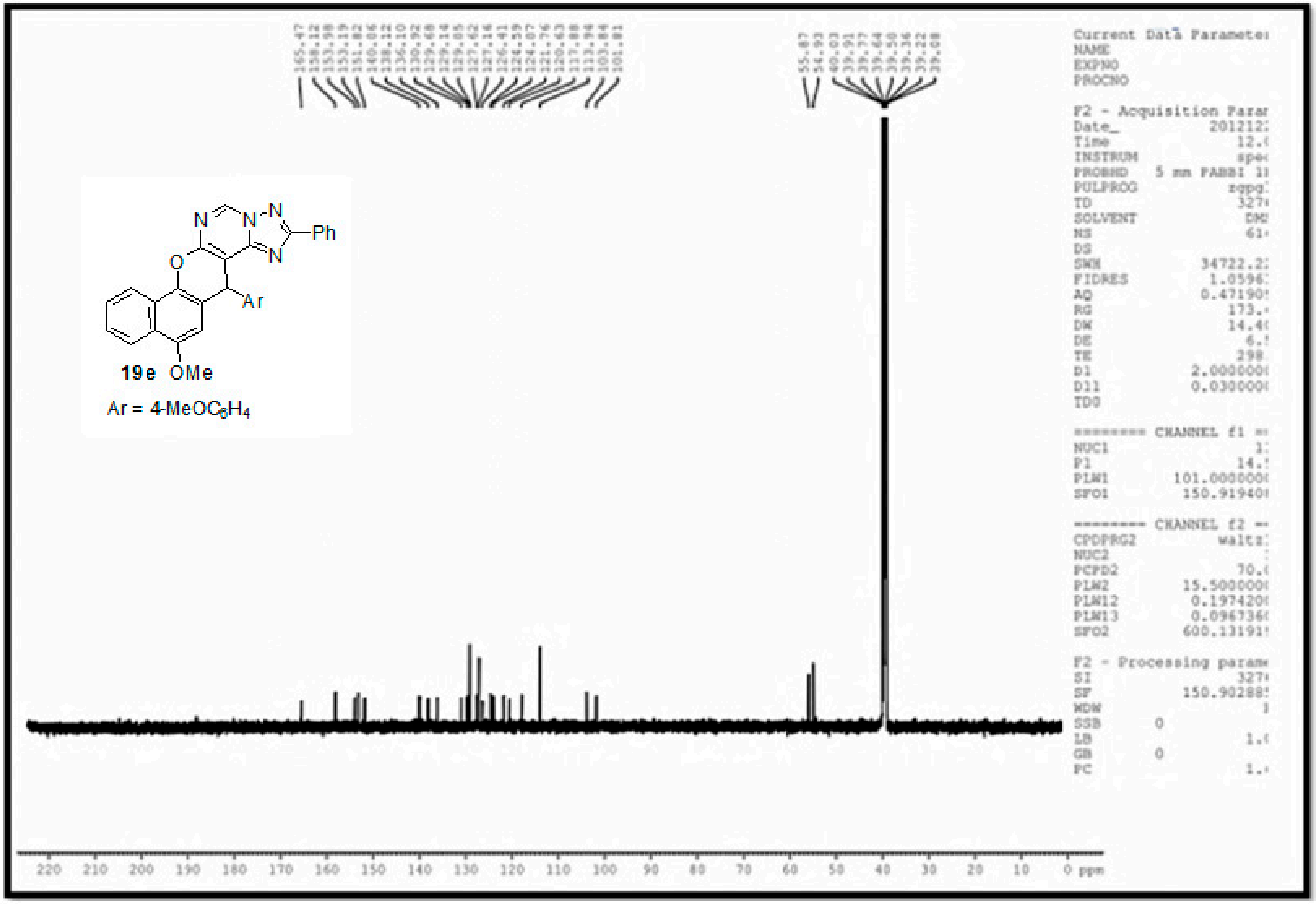
165.47
158.12
153.98
153.19
151.82
140.06
138.12
136.10
130.92
129.68
129.14
129.05
127.62
127.16
126.41
124.59
124.07
121.76
120.63
117.88
113.94
103.84
101.81
55.87
54.93
40.03
39.91
39.77
39.64
39.50
39.36
39.22
39.08
N
N
N
N
O
Ph
Ar
19e OMe
Ar = 4-MeOC6H4
Current Data Paramete
NAME
EXPNO
PROCNO
F2 - Acquisition Para
Date_ 201212
Time 12.
INSTRUM spe
PROBHD 5 mm PABBI 1
PULPROG zgpg
TD 327
SOLVENT DM
NS 61
DS
SWH 34722.2
FIDRES 1.0596
AQ 0.47190
RG 173.
DW 14.4
DE 6.
TE 298
D1 2.000000
D11 0.030000
TD0
======== CHANNEL f1 =
NUC1 1
P1 14.
PLW1 101.000000
SFO1 150.91940
======== CHANNEL f2 =
CPDPRG2 waltz
NUC2
PCPD2 70.
PLW2 15.500000
PLW12 0.197420
PLW13 0.096736
SFO2 600.13191
F2 - Processing param
SI 327
SF 150.90288
WDW
SSB 0
LB 1.
GB 0
PC 1.
220 210 200 190 180 170 160 150 140 130 120 110 100 90 80 70 60 50 40 30 20 10 0 ppm
