# Supplementary material for: Design of New Benzo[h]chromene Derivatives: Antitumor Activities and Structure-Activity Relationships of the 2,3-Positions and Fused Rings at the 2,3-Positions
Source: Molecules. 2017 Mar 18;22(3):479. doi: 10.3390/molecules22030479 (PMC6155235; doi:10.3390/molecules22030479)
Supplement: Supplementary file 1 [file molecules-22-00479-s001.zip › molecules-178589-supplementary/13C NMR of compound 4.pdf]

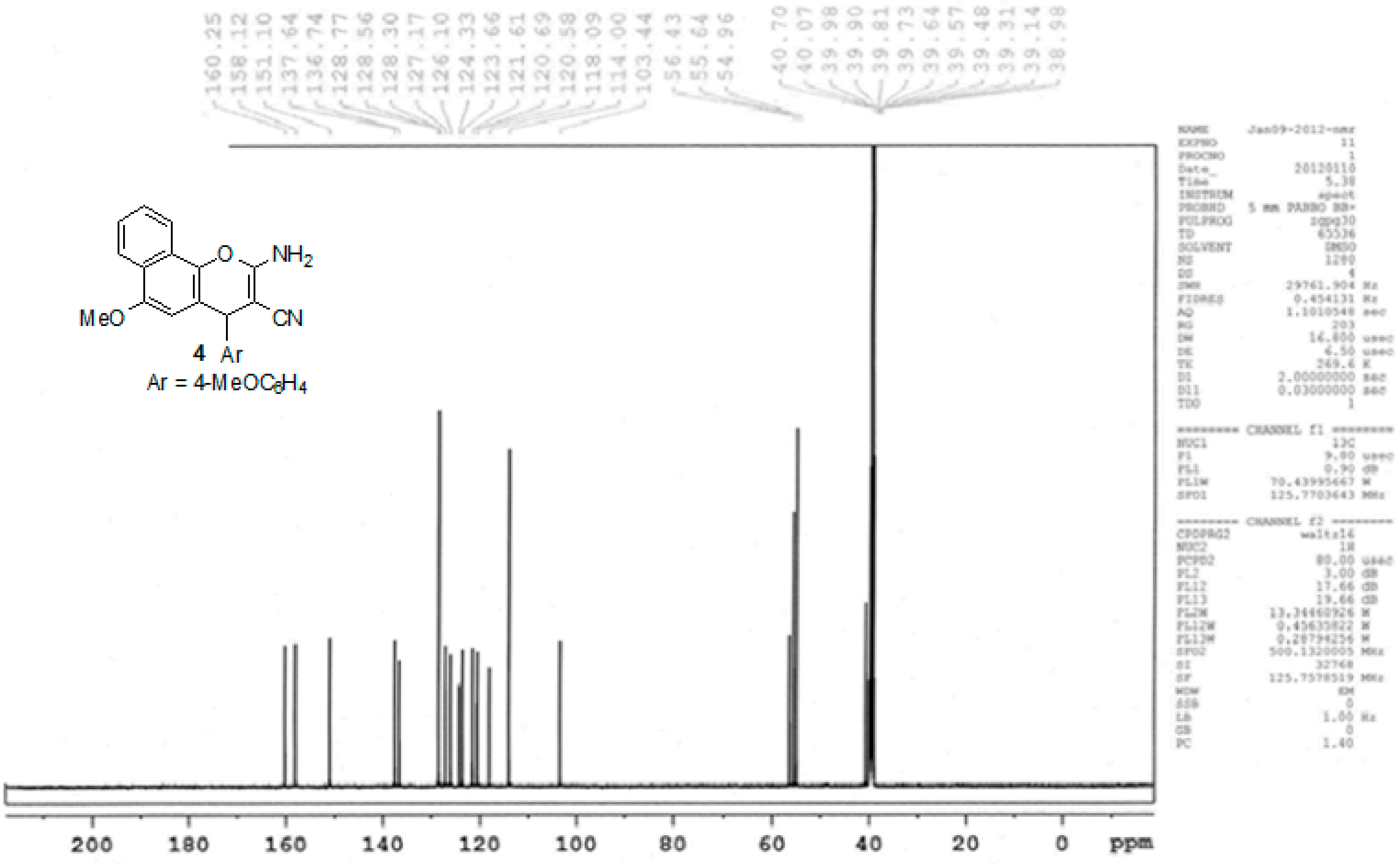
160.25
158.12
151.10
137.64
136.74
128.77
128.56
128.30
127.17
126.10
124.33
123.66
121.61
120.69
120.58
118.09
114.00
103.44
56.43
55.64
54.96
40.70
40.07
39.98
39.90
39.81
39.73
39.64
39.57
39.48
39.31
39.14
38.98
MeO
O
NH2
CN
4 Ar
Ar = 4-MeOC6H4
200 180 160 140 120 100 80 60 40 20 0 ppm
NAME Jan09-2012-nmr
EXPNO 11
PROCNO 1
Date_ 20120110
Time 5.38
INSTRUM spect
PROBHD 5 mm PABBO BB-
PULPROG zgpg30
TD 65536
SOLVENT DMSO
NS 1280
DS 4
SWH 29761.904 Hz
FIDRES 0.454131 Hz
AQ 1.1010548 sec
RG 203
DW 16.800 usec
DE 6.50 usec
TE 269.6 K
D1 2.00000000 sec
D11 0.03000000 sec
TD0 1
======== CHANNEL f1 ========
NUC1 13C
P1 9.80 usec
PL1 0.90 dB
PL1W 70.43995667 W
SFO1 125.7703643 MHz
======== CHANNEL f2 ========
CPDPRG2 waltz16
NUC2 1H
PCPD2 80.00 usec
PL2 3.00 dB
PL12 17.66 dB
PL13 19.66 dB
PL2W 13.34460926 W
PL12W 0.45635822 W
PL13W 0.28794256 W
SFO2 500.1320005 MHz
SI 32768
SF 125.7578519 MHz
WDW EM
SSB 0
LB 1.00 Hz
GB 0
PC 1.40
