# Supplementary material for: Design of New Benzo[h]chromene Derivatives: Antitumor Activities and Structure-Activity Relationships of the 2,3-Positions and Fused Rings at the 2,3-Positions
Source: Molecules. 2017 Mar 18;22(3):479. doi: 10.3390/molecules22030479 (PMC6155235; doi:10.3390/molecules22030479)
Supplement: Supplementary file 1 [file molecules-22-00479-s001.zip › molecules-178589-supplementary/13C NMR of compound 8b.pdf]

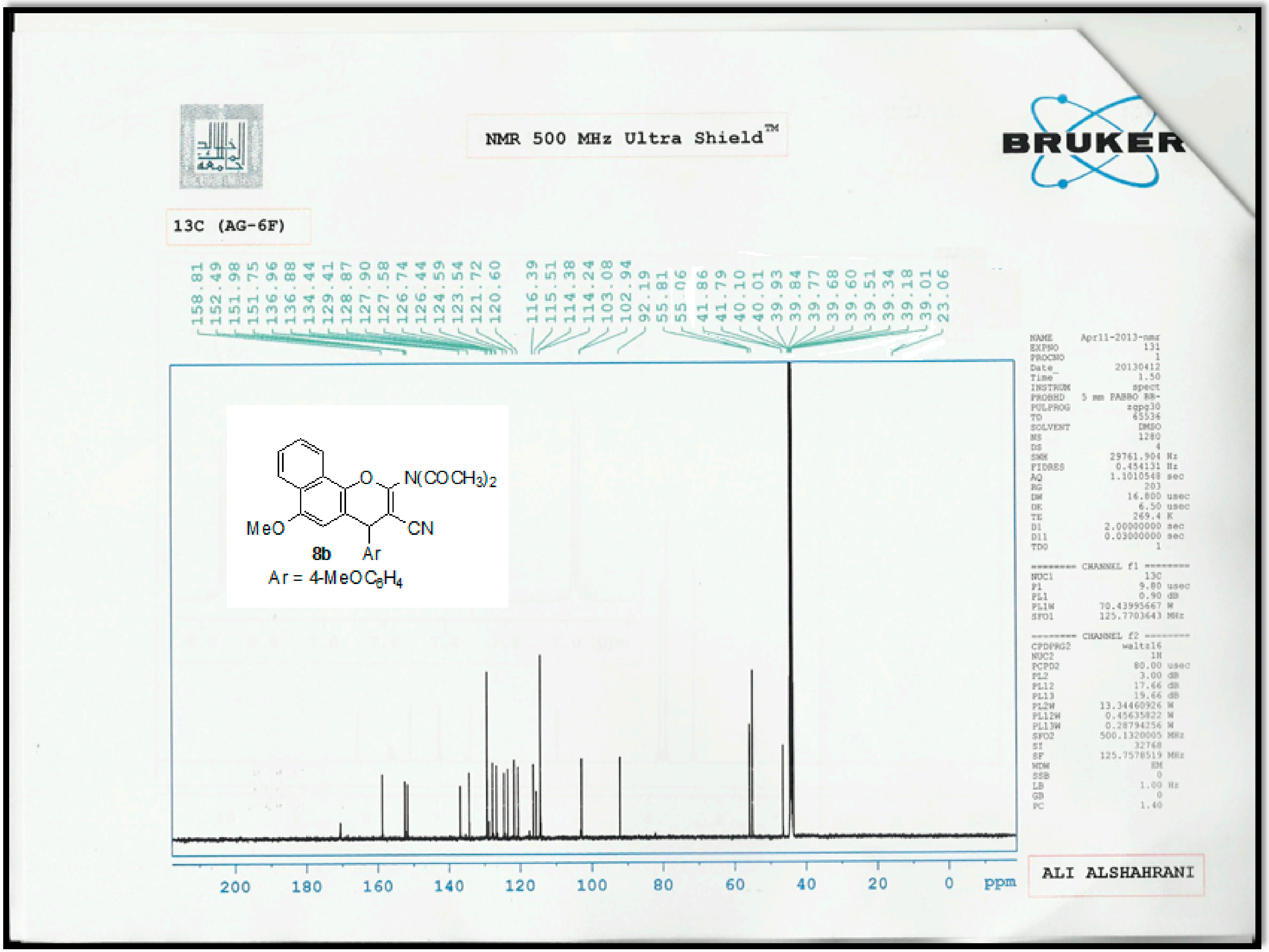

NMR 500 MHz Ultra Shield™
BRUKER
13C (AG-6F)
158.81
152.49
151.98
151.75
136.96
136.88
134.44
129.41
128.87
127.90
127.58
126.74
126.44
124.59
123.54
121.72
120.60
116.39
115.51
114.38
114.24
103.08
102.94
92.19
55.81
55.06
41.86
41.79
40.10
40.01
39.93
39.84
39.77
39.68
39.60
39.51
39.34
39.18
39.01
23.06
MeO
O
N(COCH3)2
CN
8b
Ar
Ar = 4-MeOC6H4
200
180
160
140
120
100
80
60
40
20
0
ppm
NAME April-2013-nmr
EXPNO 131
PROCNO 1
Date_ 20130412
Time 1.50
INSTRUM spect
PROBHD 5 mm PABBO BB-
PULPROG zgpg30
TD 65536
SOLVENT DMSO
NS 1280
DS 4
SWH 29761.904 Hz
FIDRES 0.454131 Hz
AQ 1.1010548 sec
RG 203
DW 16.800 usec
DE 6.50 usec
TE 269.4 K
D1 2.00000000 sec
D11 0.03000000 sec
TD0 1
======== CHANNEL f1 ========
NUC1 13C
P1 9.80 usec
PL1 0.90 dB
PL1W 70.43995667 W
SFO1 125.7703643 MHz
======== CHANNEL f2 ========
CPDPRG2 waltz16
NUC2 1H
PCPD2 80.00 usec
PL2 3.00 dB
PL12 17.46 dB
PL13 19.66 dB
PL2W 13.34460926 W
PL12W 0.45635822 W
PL13W 0.28794256 W
SFO2 500.1320005 MHz
SI 32768
SF 125.7578519 MHz
WDW EM
SSB 0
LB 1.00 Hz
GB 0
PC 1.40
ALI ALSHAHRANI
