# Supplementary material for: Design of New Benzo[h]chromene Derivatives: Antitumor Activities and Structure-Activity Relationships of the 2,3-Positions and Fused Rings at the 2,3-Positions
Source: Molecules. 2017 Mar 18;22(3):479. doi: 10.3390/molecules22030479 (PMC6155235; doi:10.3390/molecules22030479)
Supplement: Supplementary file 1 [file molecules-22-00479-s001.zip › molecules-178589-supplementary/1H NMR + D2O of compound 4.pdf]

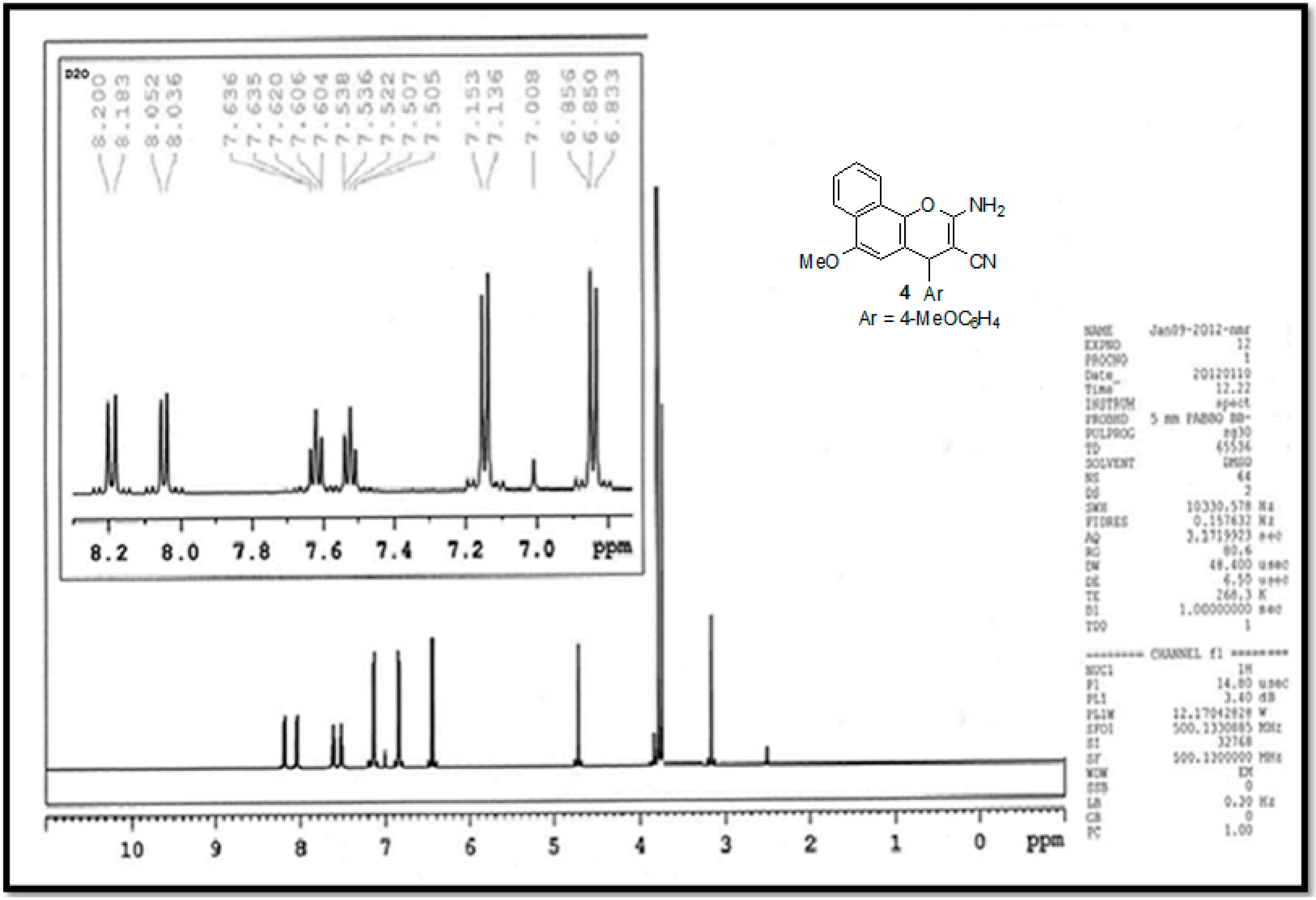
D2O
8.200
8.183
8.052
8.036
7.636
7.635
7.620
7.606
7.604
7.538
7.536
7.522
7.507
7.505
7.153
7.136
7.008
6.856
6.850
6.833
MeO
O
NH2
CN
4 Ar
Ar = 4-MeOC6H4
NAME Jan09-2012-nmr
EXPNO 12
PROCNO 1
Date_ 20120110
Time 12.22
INSTRUM spect
PULPROG zg30
TD 65536
SOLVENT DMSO
NS 64
DS 2
SWH 10330.578 Hz
FIDRES 0.157632 Hz
AQ 3.1719923 sec
RG 80.6
DW 48.400 usec
DE 6.50 usec
TE 268.3 K
D1 1.00000000 sec
TD0 1
======== CHANNEL f1 ========
NUC1 1H
P1 14.80 usec
PL1 3.40 dB
PL1W 12.17042828 W
SFO1 500.1330885 MHz
SI 32768
SF 500.1300000 MHz
WDW EM
SSB 0
LB 0.30 Hz
GB 0
PC 1.00
ppm
