# Supplementary material for: Design of New Benzo[h]chromene Derivatives: Antitumor Activities and Structure-Activity Relationships of the 2,3-Positions and Fused Rings at the 2,3-Positions
Source: Molecules. 2017 Mar 18;22(3):479. doi: 10.3390/molecules22030479 (PMC6155235; doi:10.3390/molecules22030479)
Supplement: Supplementary file 1 [file molecules-22-00479-s001.zip › molecules-178589-supplementary/1H NMR 8-4 ppm + D2O of compound 6.pdf]

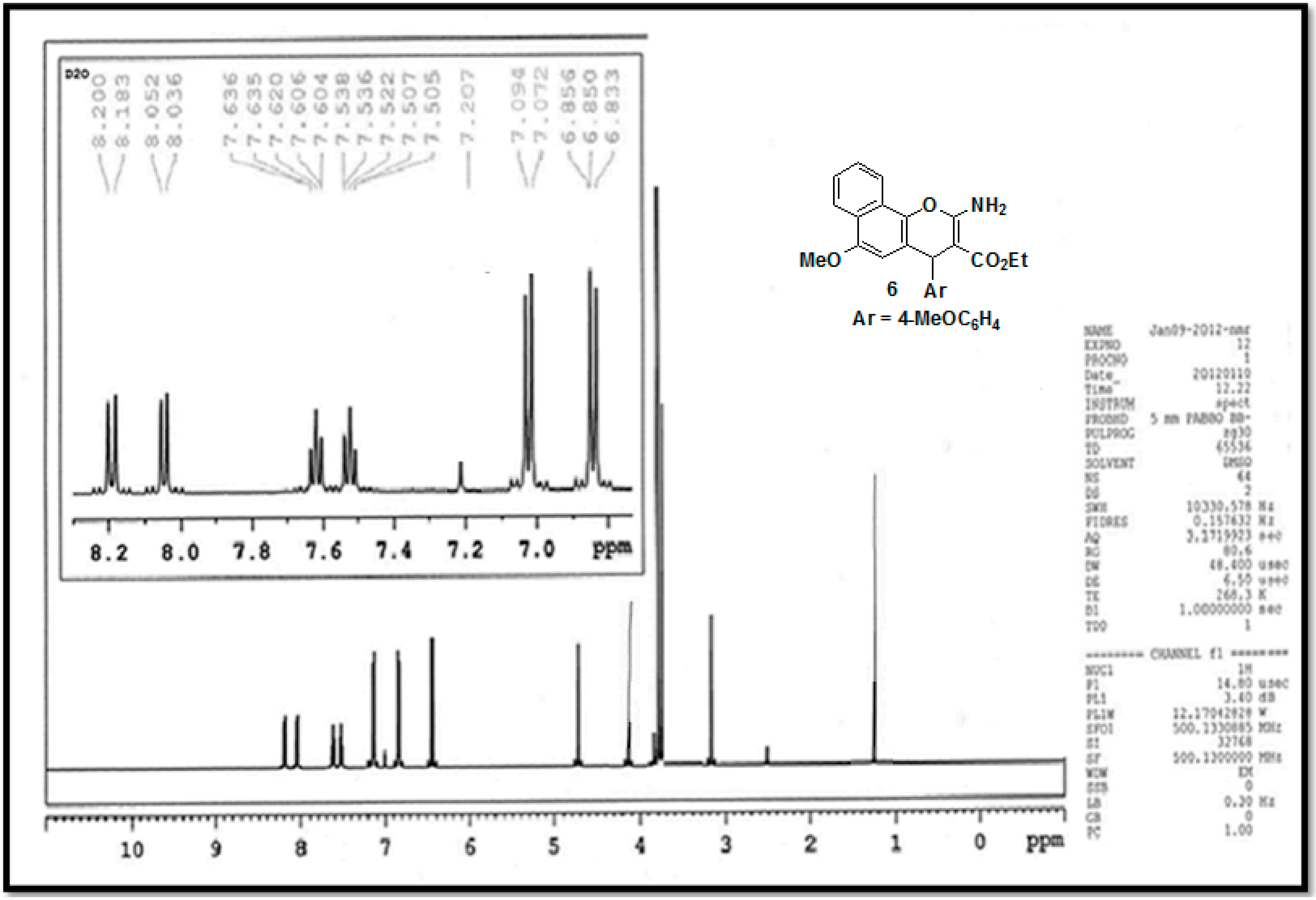

D2O
8.200
8.183
8.052
8.036
7.636
7.635
7.620
7.606
7.604
7.538
7.536
7.522
7.507
7.505
7.207
7.094
7.072
6.856
6.850
6.833
8.2 8.0 7.8 7.6 7.4 7.2 7.0 ppm
MeO
O
NH2
CO2Et
6
Ar
Ar = 4-MeOC6H4
10 9 8 7 6 5 4 3 2 1 0 ppm
