# Supplementary material for: Design of New Benzo[h]chromene Derivatives: Antitumor Activities and Structure-Activity Relationships of the 2,3-Positions and Fused Rings at the 2,3-Positions
Source: Molecules. 2017 Mar 18;22(3):479. doi: 10.3390/molecules22030479 (PMC6155235; doi:10.3390/molecules22030479)
Supplement: Supplementary file 1 [file molecules-22-00479-s001.zip › molecules-178589-supplementary/1H NMR 8-4 ppm of compound 4.pdf]

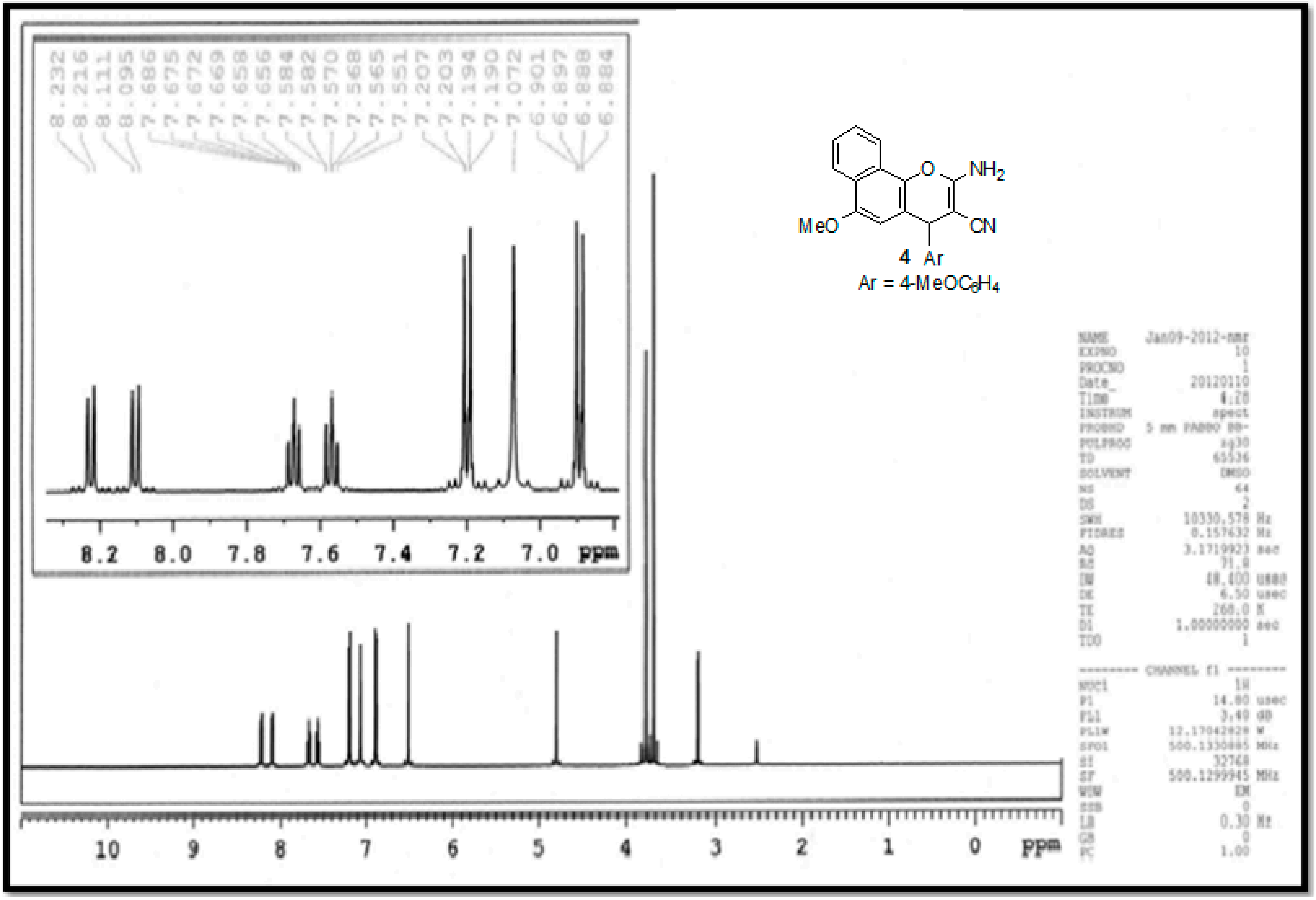
8.232
8.216
8.111
8.095
7.686
7.675
7.672
7.669
7.658
7.656
7.584
7.582
7.570
7.568
7.565
7.551
7.207
7.203
7.194
7.190
7.072
6.901
6.897
6.888
6.884
8.2
8.0
7.8
7.6
7.4
7.2
7.0
ppm
MeO
O
NH2
CN
4
Ar
Ar = 4-MeOC6H4
10
9
8
7
6
5
4
3
2
1
0
ppm
NAME Jan09-2012-nmr
EXPNO 10
PROCNO 1
Date_ 20120110
INSTRUM spect
SOLVENT DMSO
NS 64
DS 2
SWH 10330.578 Hz
AQ 3.1719923 sec
DE 6.50 usec
D1 1.00000000 sec
TD0 1
CHANNEL f1
NUC1 1H
P1 14.80 usec
SF 500.1299945 MHz
LB 0.30 Hz
PC 1.00
