# Supplementary material for: Design of New Benzo[h]chromene Derivatives: Antitumor Activities and Structure-Activity Relationships of the 2,3-Positions and Fused Rings at the 2,3-Positions
Source: Molecules. 2017 Mar 18;22(3):479. doi: 10.3390/molecules22030479 (PMC6155235; doi:10.3390/molecules22030479)
Supplement: Supplementary file 1 [file molecules-22-00479-s001.zip › molecules-178589-supplementary/1H NMR 8-4 ppm of compound 8b.pdf]

NMR 500 MHz Ultra Shield™

1H (AG-6F)

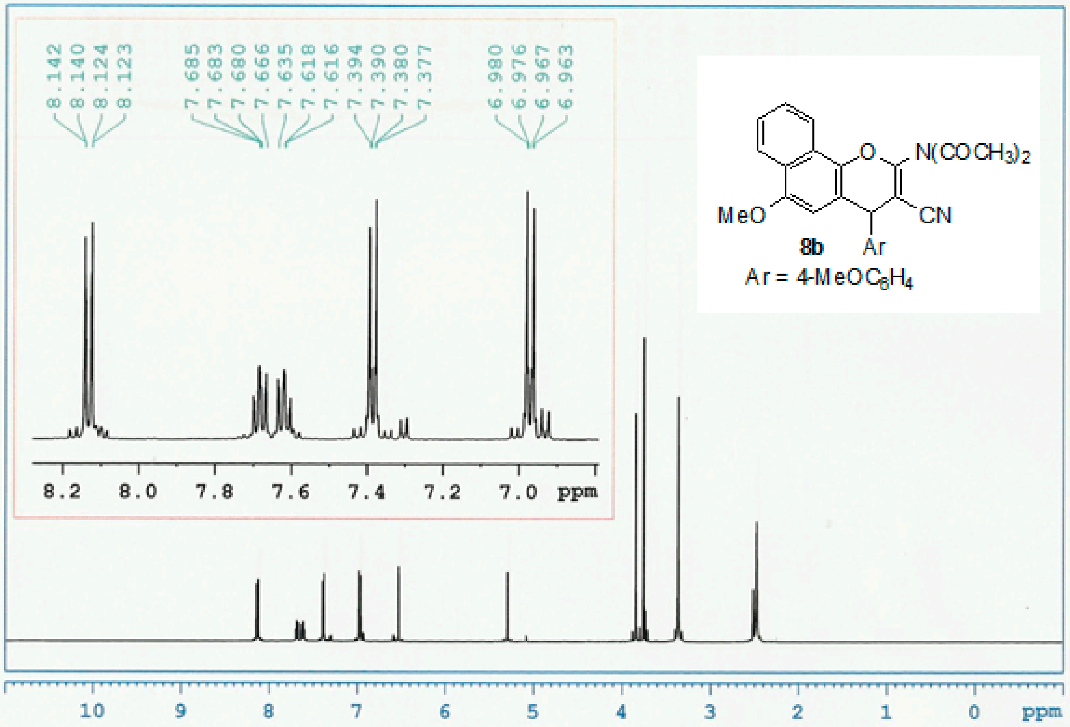

NAME	Apr11-2013-nmr	
EXPNO	130	
PROCNO	1	
Date_	20130412	
Time	0.40	
INSTRUM	spect	
PROBHD	5 mm PABBO BB-	
PULPROG	zg30	
TD	65536	
SOLVENT	DMSO	
NS	64	
DS	2	
SWH	10330.578	Hz
FIDRES	0.157632	Hz
AQ	3.1719923	sec
RG	114	
DW	48.400	usec
DE	6.50	usec
TE	267.7	K
D1	1.00000000	sec
TD0	1	
======== CHANNEL f1 ========		
NUC1	1H	
P1	14.80	usec
PL1	3.40	dB
PL1W	12.17042828	W
SFO1	500.1330885	MHz
SI	32768	
SF	500.1300000	MHz
WDW	EM	
SSB	0	
LB	0.30	Hz
GB	0	
PC	1.00	

ALI ALSHAHRANI
