# Supplementary material for: Design of New Benzo[h]chromene Derivatives: Antitumor Activities and Structure-Activity Relationships of the 2,3-Positions and Fused Rings at the 2,3-Positions
Source: Molecules. 2017 Mar 18;22(3):479. doi: 10.3390/molecules22030479 (PMC6155235; doi:10.3390/molecules22030479)
Supplement: Supplementary file 1 [file molecules-22-00479-s001.zip › molecules-178589-supplementary/1H NMR of compound 16.pdf]

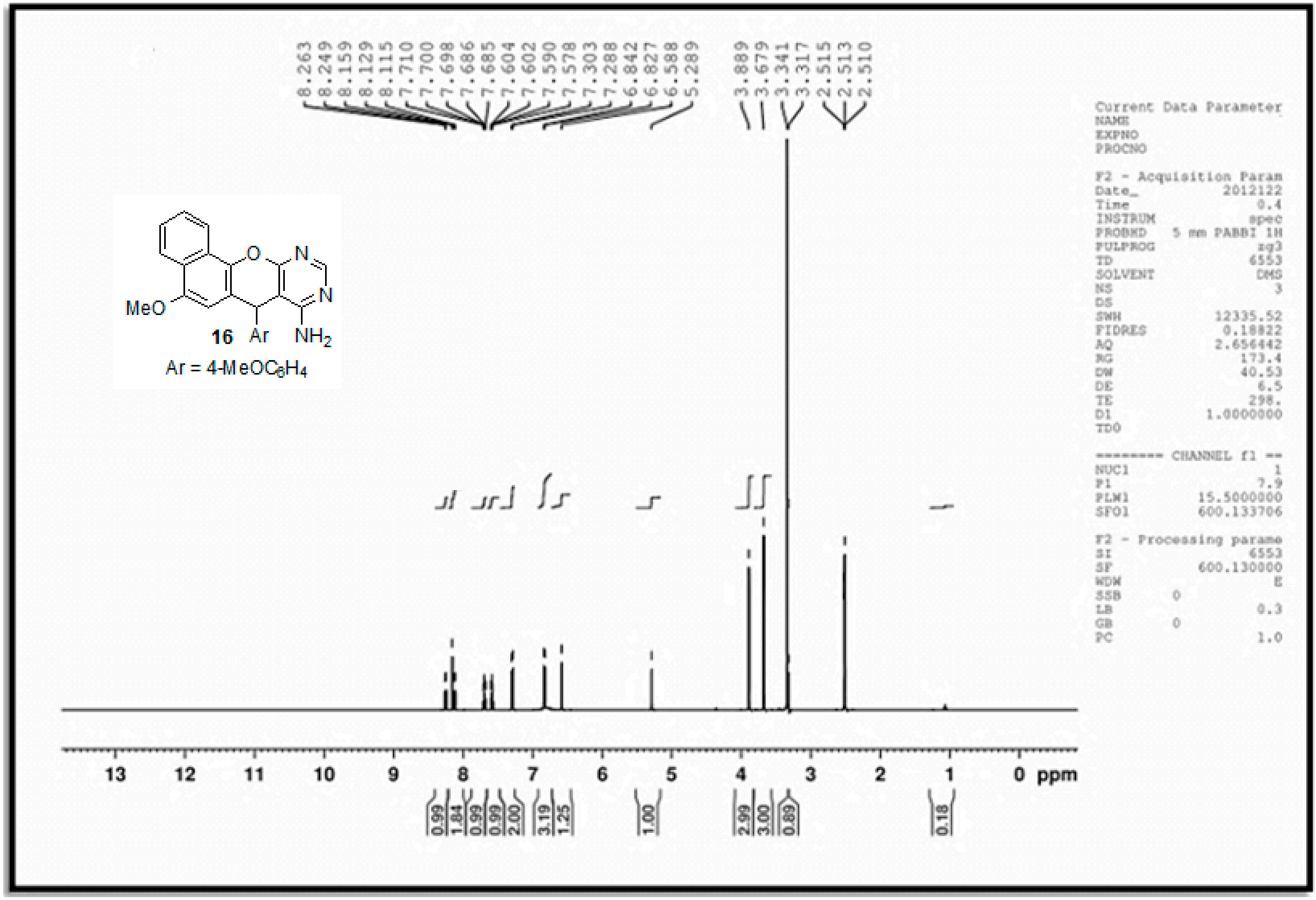

MeO
16
Ar
NH2
Ar = 4-MeOC6H4
8.263 8.249 8.159 8.129 8.115 7.710 7.700 7.698 7.686 7.685 7.604 7.602 7.590 7.578 7.303 7.288 6.842 6.827 6.588 5.289 3.889 3.679 3.341 3.317 2.515 2.513 2.510
Current Data Parameter
NAME
EXPNO
PROCNO
F2 - Acquisition Param
Date_ 2012122
Time 0.4
INSTRUM spec
PROBHD 5 mm PABBI 1H
PULPROG zg3
TD 6553
SOLVENT DMS
NS 3
DS
SWH 12335.52
FIDRES 0.18822
AQ 2.656442
RG 173.4
DW 40.53
DE 6.5
TE 298.
D1 1.0000000
TD0
======== CHANNEL f1 ==
NUC1 1
P1 7.9
PLW1 15.5000000
SFO1 600.133706
F2 - Processing parame
SI 6553
SF 600.130000
WDW E
SSB 0
LB 0.3
GB 0
PC 1.0
13 12 11 10 9 8 7 6 5 4 3 2 1 0 ppm
0.99 1.84 0.99 0.99 2.00 3.19 1.25 1.00 2.99 3.00 0.89 0.18
