# Supplementary material for: Design of New Benzo[h]chromene Derivatives: Antitumor Activities and Structure-Activity Relationships of the 2,3-Positions and Fused Rings at the 2,3-Positions
Source: Molecules. 2017 Mar 18;22(3):479. doi: 10.3390/molecules22030479 (PMC6155235; doi:10.3390/molecules22030479)
Supplement: Supplementary file 1 [file molecules-22-00479-s001.zip › molecules-178589-supplementary/1H NMR of compound 8b.pdf]

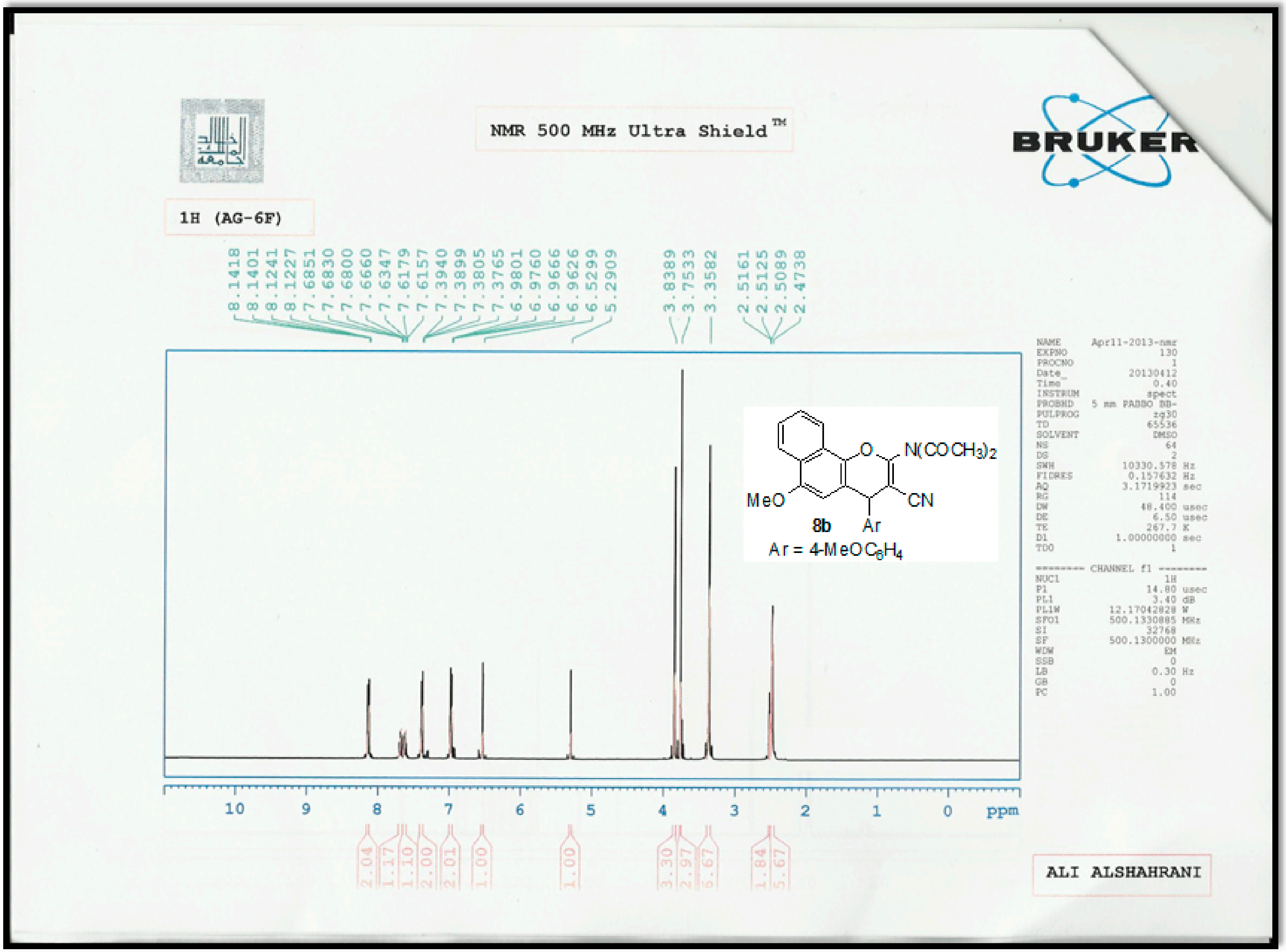
NMR 500 MHz Ultra Shield™
1H (AG-6F)
BRUKER
8.1418
8.1401
8.1241
8.1227
7.6851
7.6830
7.6800
7.6660
7.6347
7.6179
7.6157
7.3940
7.3899
7.3805
7.3765
6.9801
6.9760
6.9666
6.9626
6.5299
5.2909
3.8389
3.7533
3.3582
2.5161
2.5125
2.5089
2.4738
MeO
O
N(COCH3)2
CN
8b
Ar
Ar = 4-MeOC6H4
10
9
8
7
6
5
4
3
2
1
0
ppm
2.04
1.17
1.10
2.00
2.01
1.00
1.00
3.30
2.97
6.67
1.84
5.67
NAME April-2013-nmr
EXPNO 130
PROCNO 1
Date_ 20130412
Time 0.40
INSTRUM spect
PROBHD 5 mm PABBO BB-
PULPROG zg30
TD 65536
SOLVENT DMSO
NS 64
DS 2
SWH 10330.578 Hz
FIDRES 0.157632 Hz
AQ 3.1719923 sec
RG 114
DW 48.400 usec
DE 6.50 usec
TE 267.7 K
D1 1.00000000 sec
TD0 1
======== CHANNEL f1 ========
NUC1 1H
P1 14.80 usec
PL1 3.40 dB
PL1W 12.17042828 W
SFO1 500.1330885 MHz
SI 32768
SF 500.1300000 MHz
WDW EM
SSB 0
LB 0.30 Hz
GB 0
PC 1.00
ALI ALSHAHRANI
