# Supplementary material for: Design of New Benzo[h]chromene Derivatives: Antitumor Activities and Structure-Activity Relationships of the 2,3-Positions and Fused Rings at the 2,3-Positions
Source: Molecules. 2017 Mar 18;22(3):479. doi: 10.3390/molecules22030479 (PMC6155235; doi:10.3390/molecules22030479)
Supplement: Supplementary file 1 [file molecules-22-00479-s001.zip › molecules-178589-supplementary/1H NMR of compound 9.pdf]

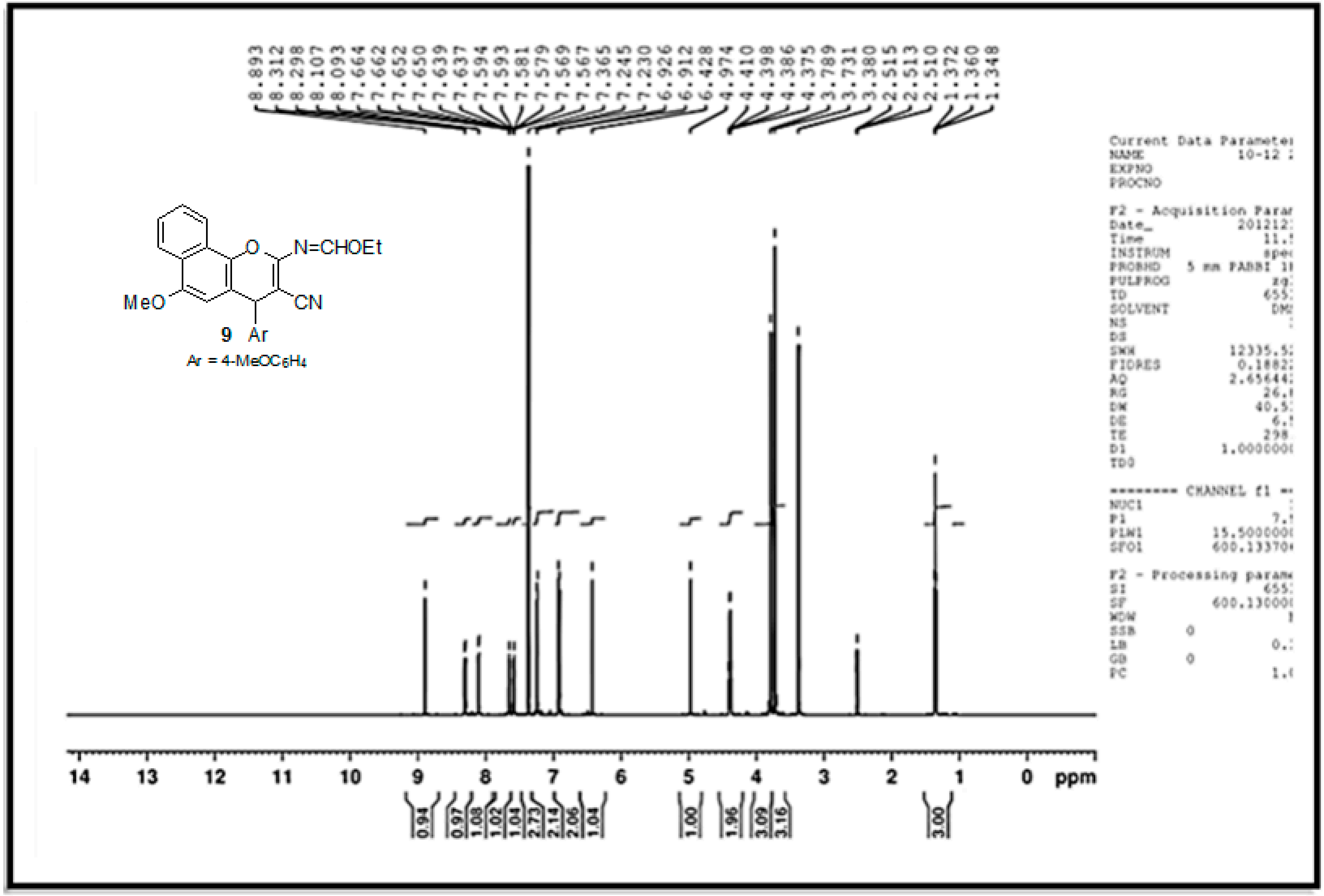
N=CHOEt
CN
MeO
O
9 Ar
Ar = 4-MeOC6H4
8.893 8.312 8.298 8.107 8.093 7.664 7.662 7.652 7.650 7.639 7.637 7.594 7.593 7.581 7.579 7.569 7.567 7.365 7.245 7.230 6.926 6.912 6.428 4.974 4.410 4.398 4.386 4.375 3.789 3.731 3.380 2.515 2.513 2.510 1.372 1.360 1.348
Current Data Parameter
NAME 10-12
EXPNO
PROCNO
F2 - Acquisition Param
Date_ 201212
Time 11.
INSTRUM spec
PROBHD 5 mm PABBI 1H
PULPROG zg
TD 655
SOLVENT DM
NS
DS
SWH 12335.5
FIDRES 0.1882
AQ 2.65644
RG 26.
DW 40.5
DE 6.
TE 298
D1 1.000000
TD0
======== CHANNEL f1 ==
NUC1
P1 7.
PLW1 15.500000
SFO1 600.13370
F2 - Processing param
SI 655
SF 600.13000
WDW
SSB 0
LB 0.
GB 0
PC 1.
14 13 12 11 10 9 8 7 6 5 4 3 2 1 0 ppm
0.94 0.97 1.08 1.02 1.04 2.73 2.14 2.06 1.04 1.00 1.96 3.09 3.16 3.00
