# Supplementary figures and images for: Design of New Benzo[h]chromene Derivatives: Antitumor Activities and Structure-Activity Relationships of the 2,3-Positions and Fused Rings at the 2,3-Positions
Source: Molecules. 2017 Mar 18;22(3):479. doi: 10.3390/molecules22030479 (PMC6155235; doi:10.3390/molecules22030479)

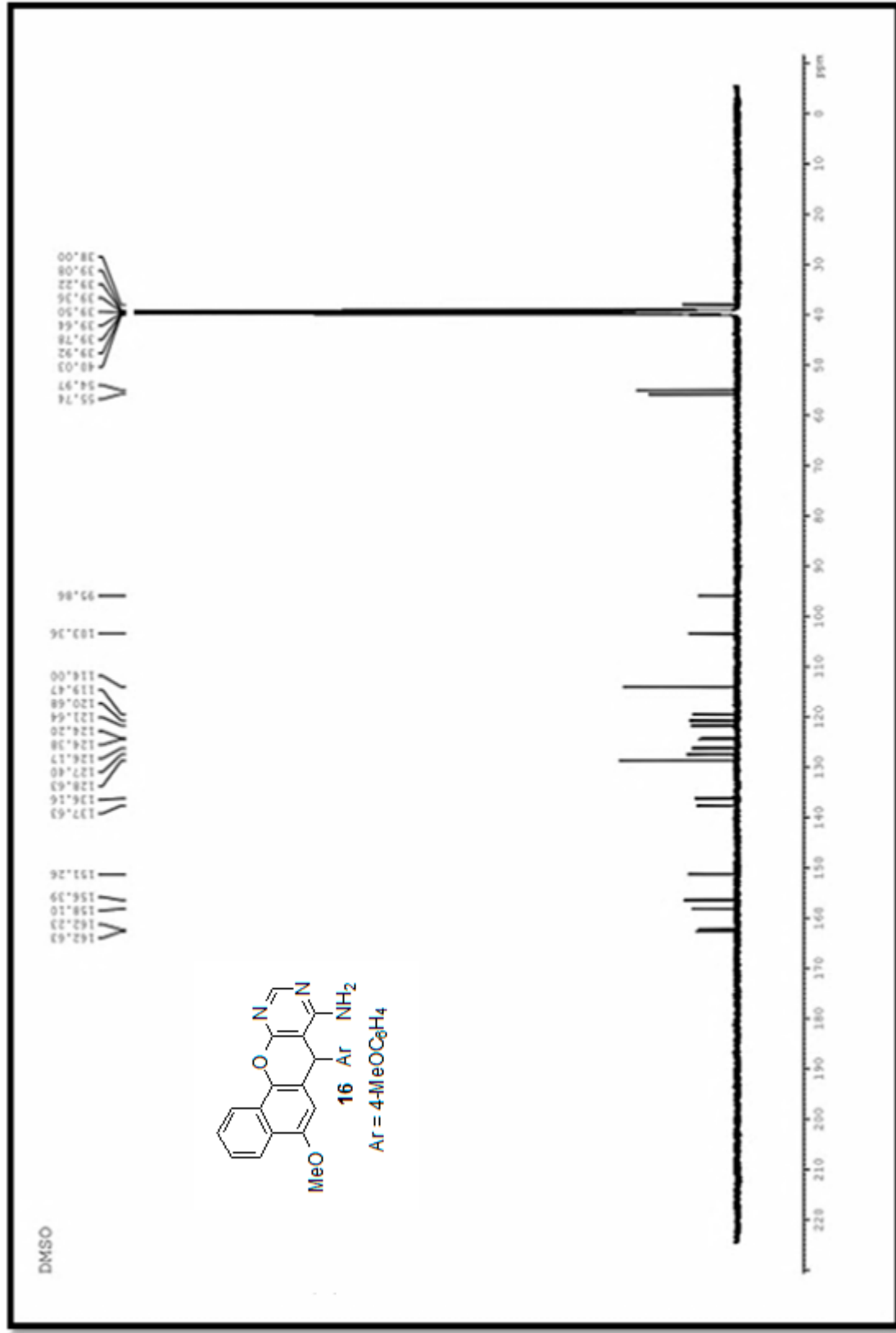

Supplement: Supplementary file 1 [file molecules-22-00479-s001.zip › molecules-178589-supplementary/13C NMR of compound 16 (1).pdf]

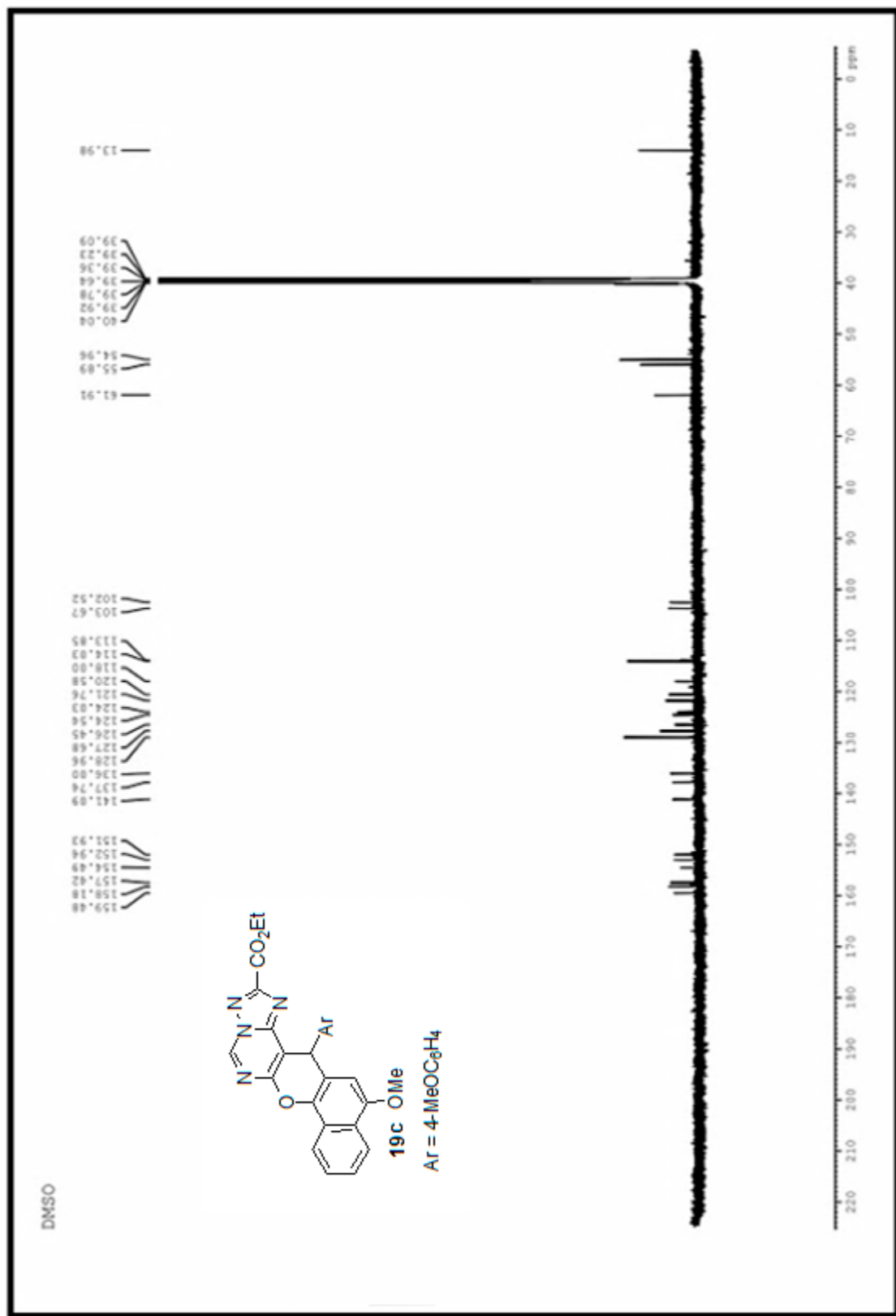

Supplement: Supplementary file 1 [file molecules-22-00479-s001.zip › molecules-178589-supplementary/13C NMR of compound 19c.pdf]

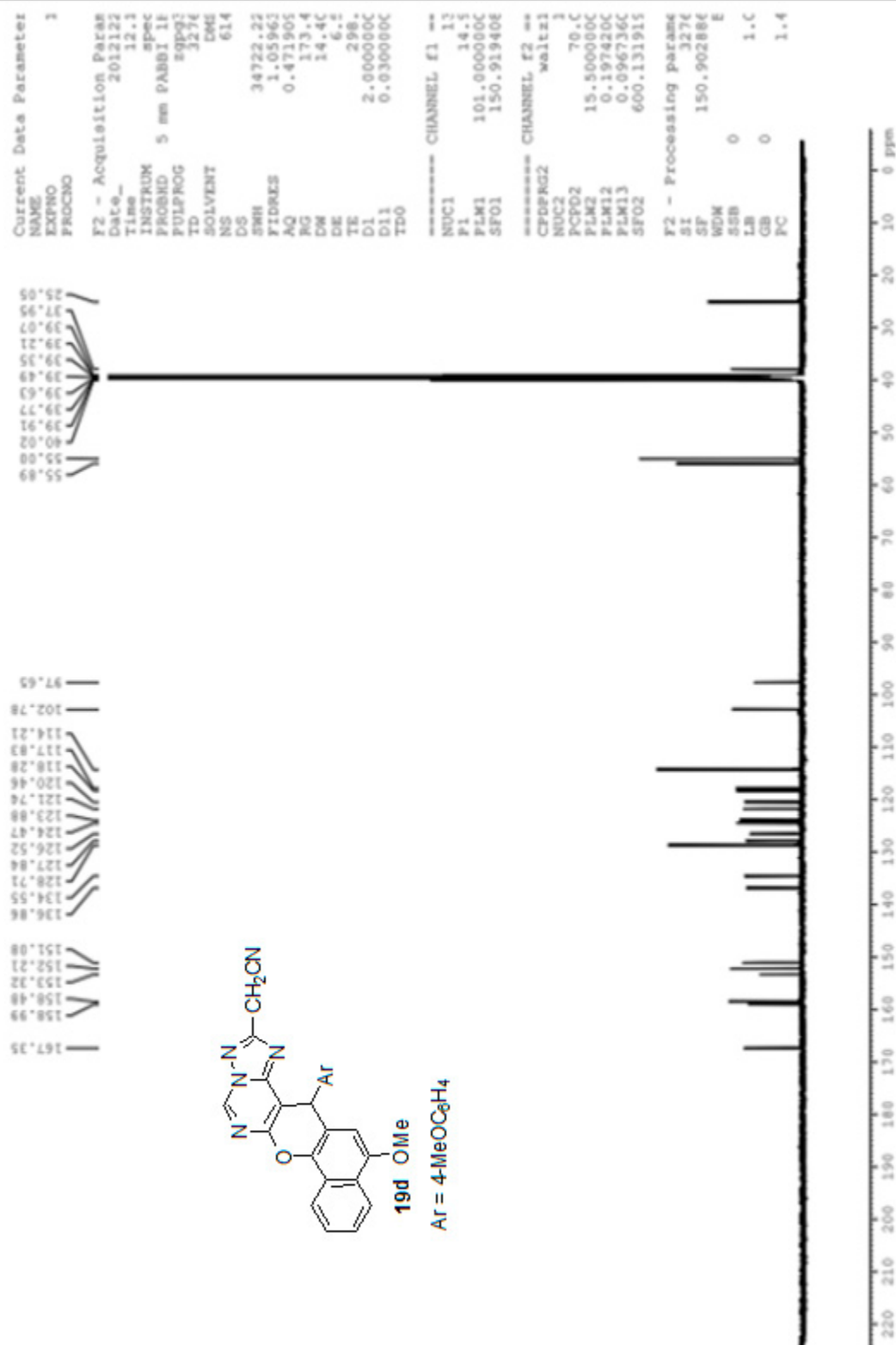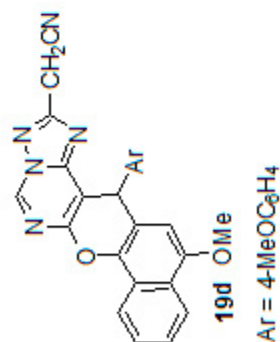

Supplement: Supplementary file 1 [file molecules-22-00479-s001.zip › molecules-178589-supplementary/13C NMR of compound 19d.pdf]

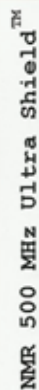

NMR 500 MHz Ultra Shield™

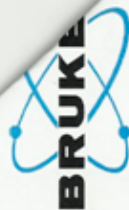

13C (AG-5F)

[illegible]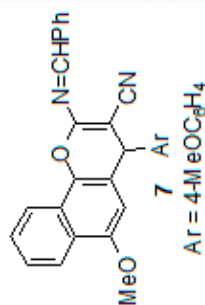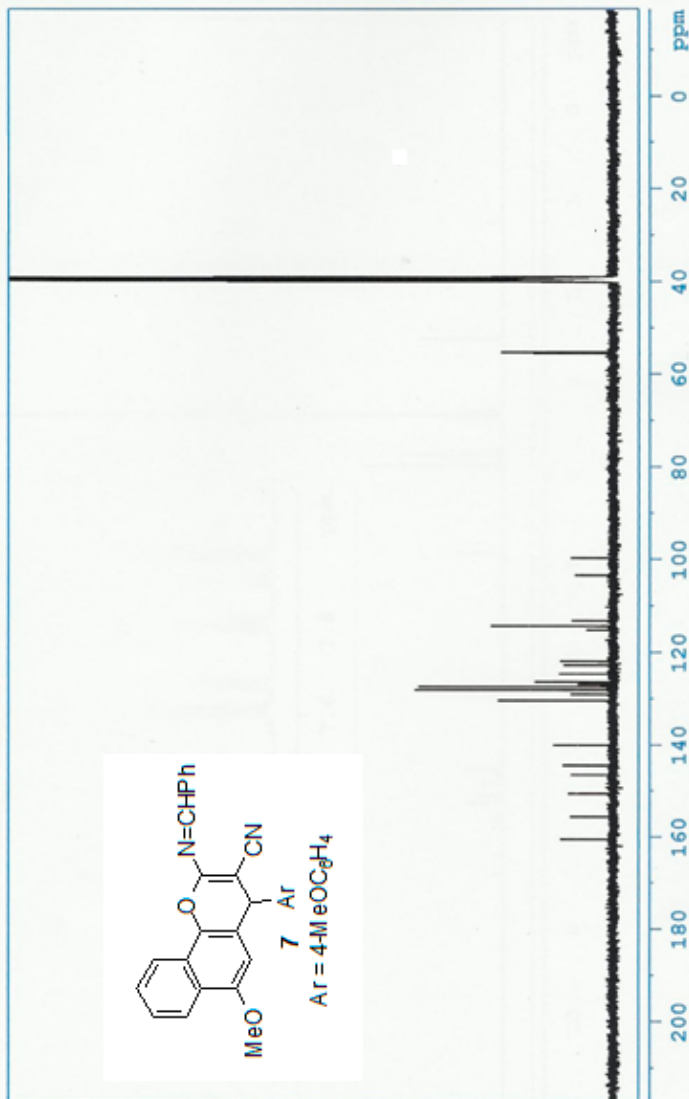[illegible]

ALI ALSHAHRANI

Supplement: Supplementary file 1 [file molecules-22-00479-s001.zip › molecules-178589-supplementary/13C NMR of compound 7.pdf]

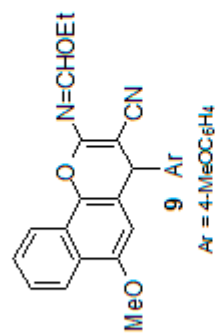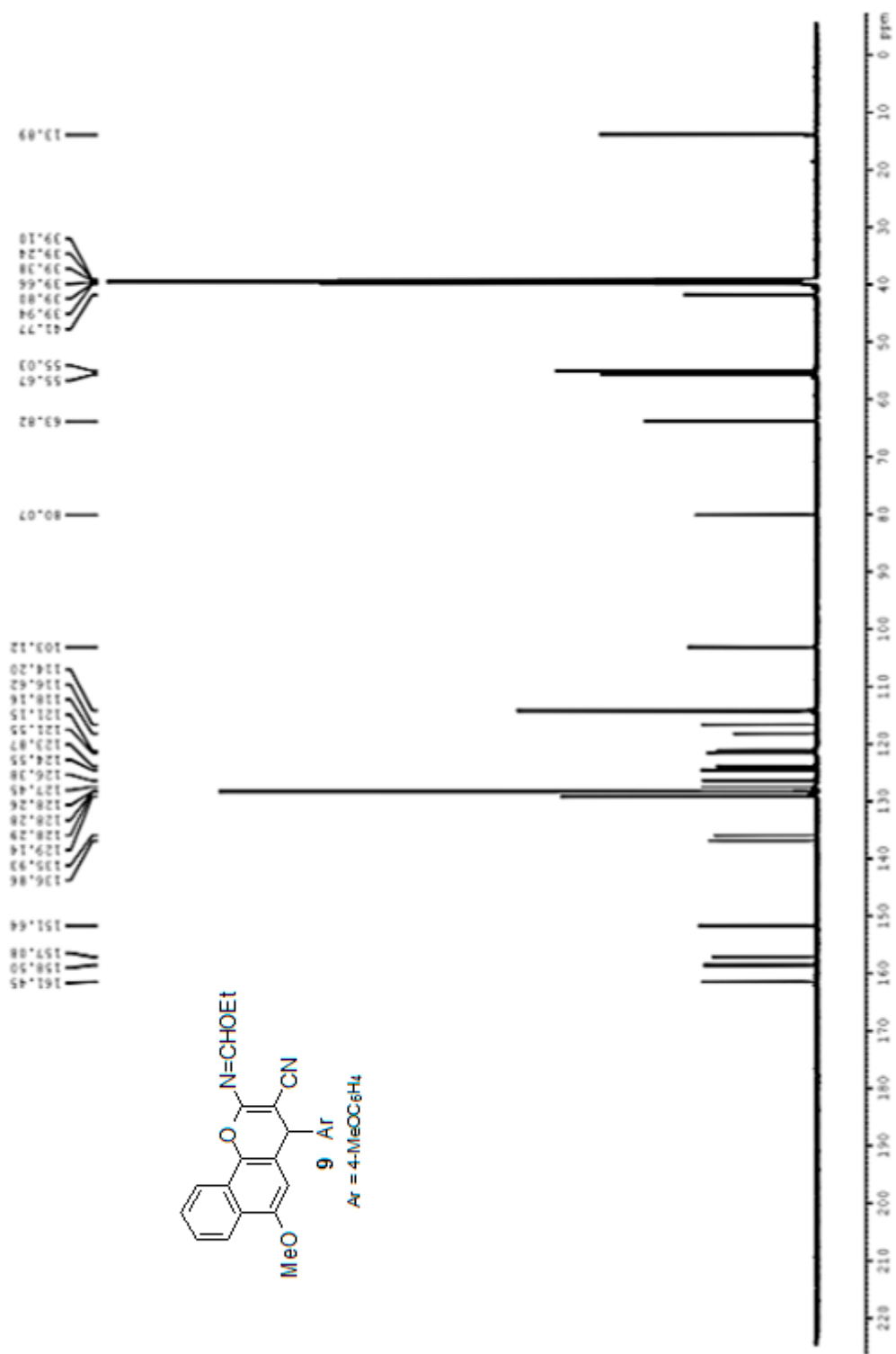

Supplement: Supplementary file 1 [file molecules-22-00479-s001.zip › molecules-178589-supplementary/13C NMR of compound 9.pdf]

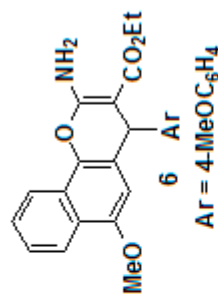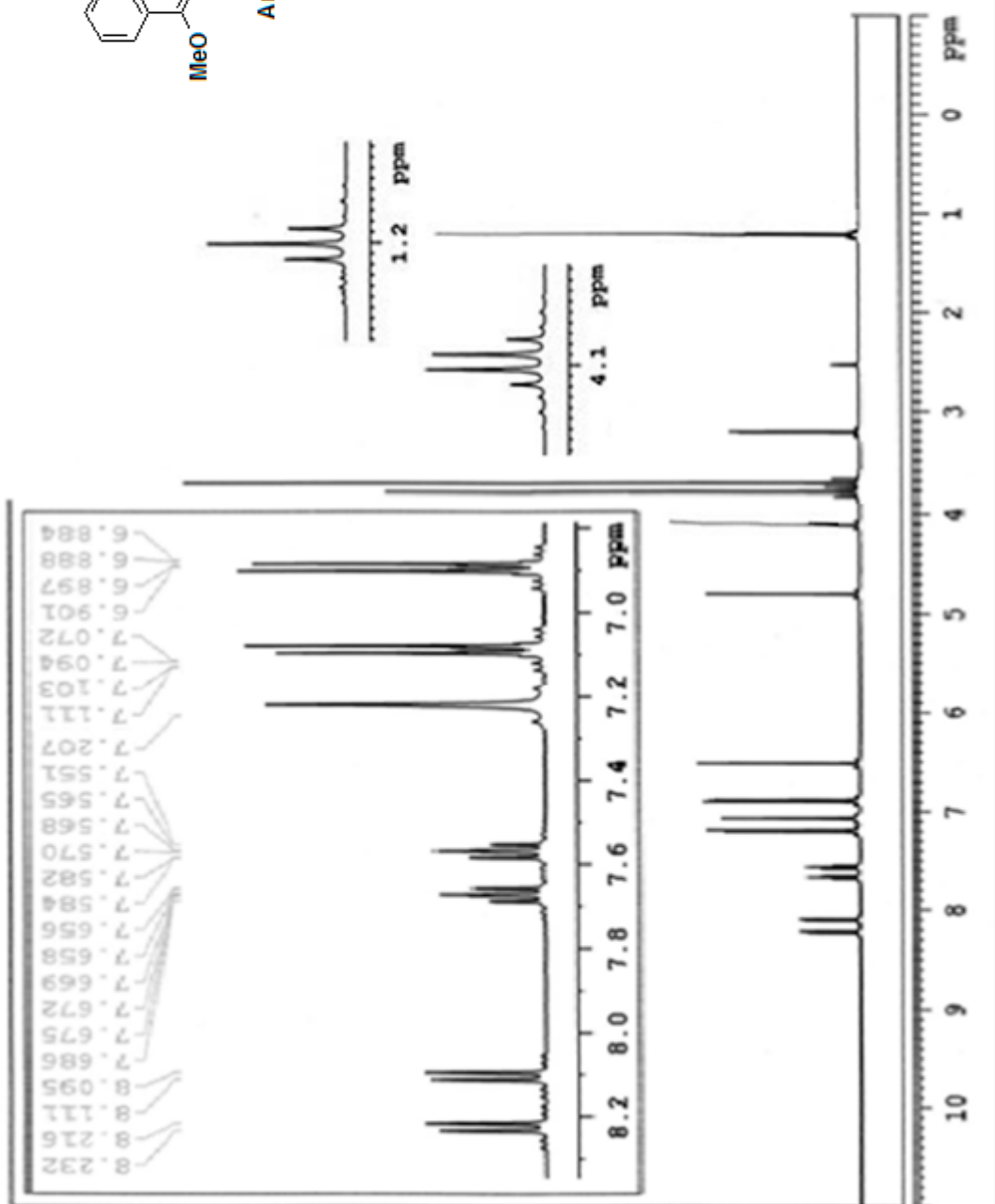

Supplement: Supplementary file 1 [file molecules-22-00479-s001.zip › molecules-178589-supplementary/1H NMR 8-4 ppm of compound 6.pdf]

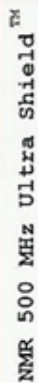

NMR 500 MHz Ultra Shield™

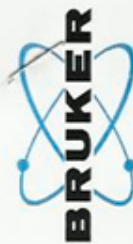

1H (AG-5F)

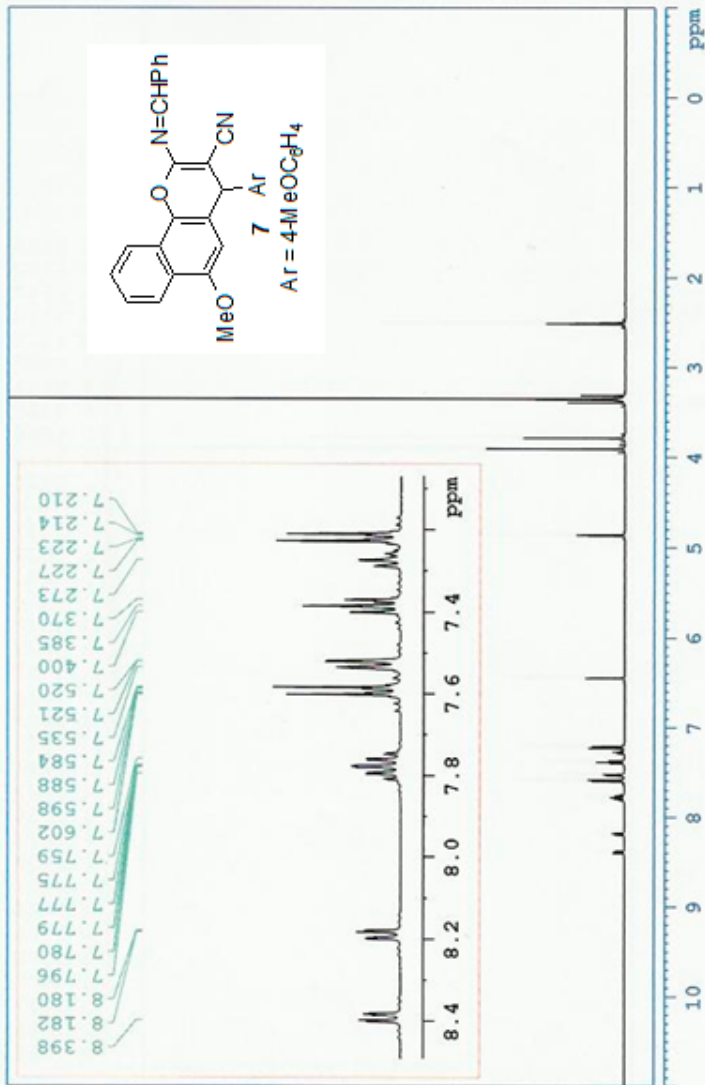[illegible]

ALI ALSHAHRANI

Supplement: Supplementary file 1 [file molecules-22-00479-s001.zip › molecules-178589-supplementary/1H NMR 8-4 ppm of compound 7.pdf]

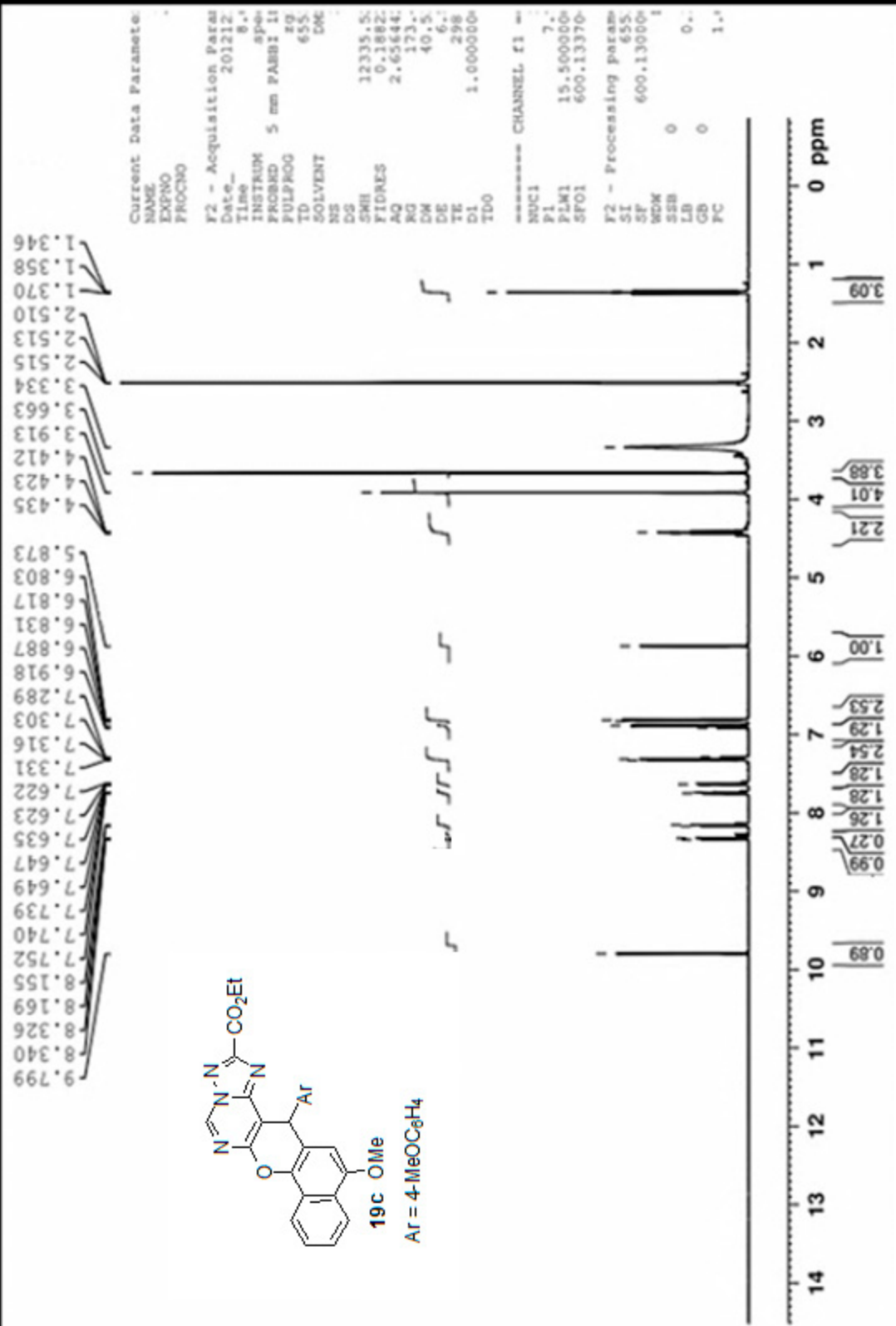

Supplement: Supplementary file 1 [file molecules-22-00479-s001.zip › molecules-178589-supplementary/1H NMR of compound 19c.pdf]

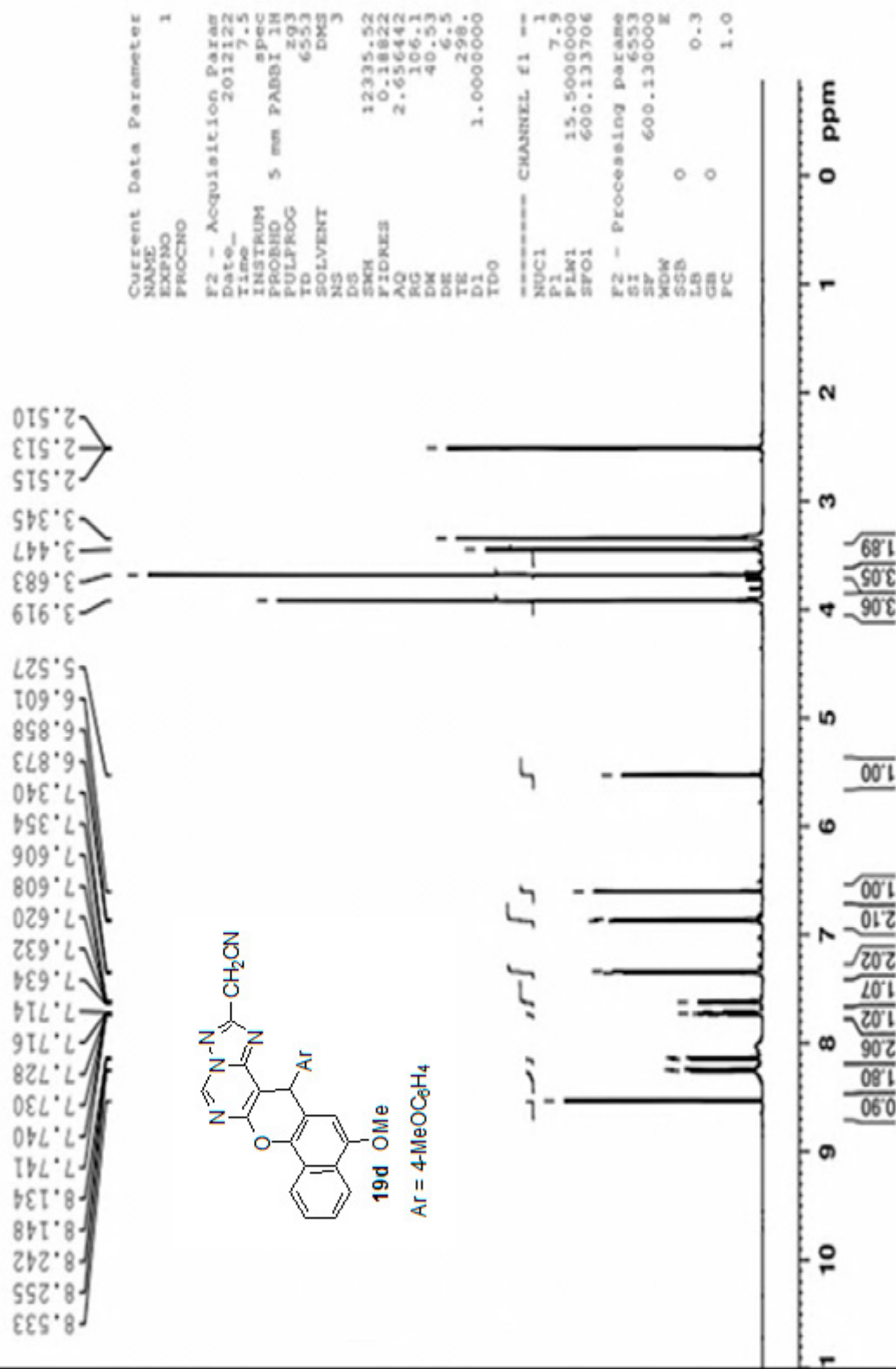

Supplement: Supplementary file 1 [file molecules-22-00479-s001.zip › molecules-178589-supplementary/1H NMR of compound 19d.pdf]

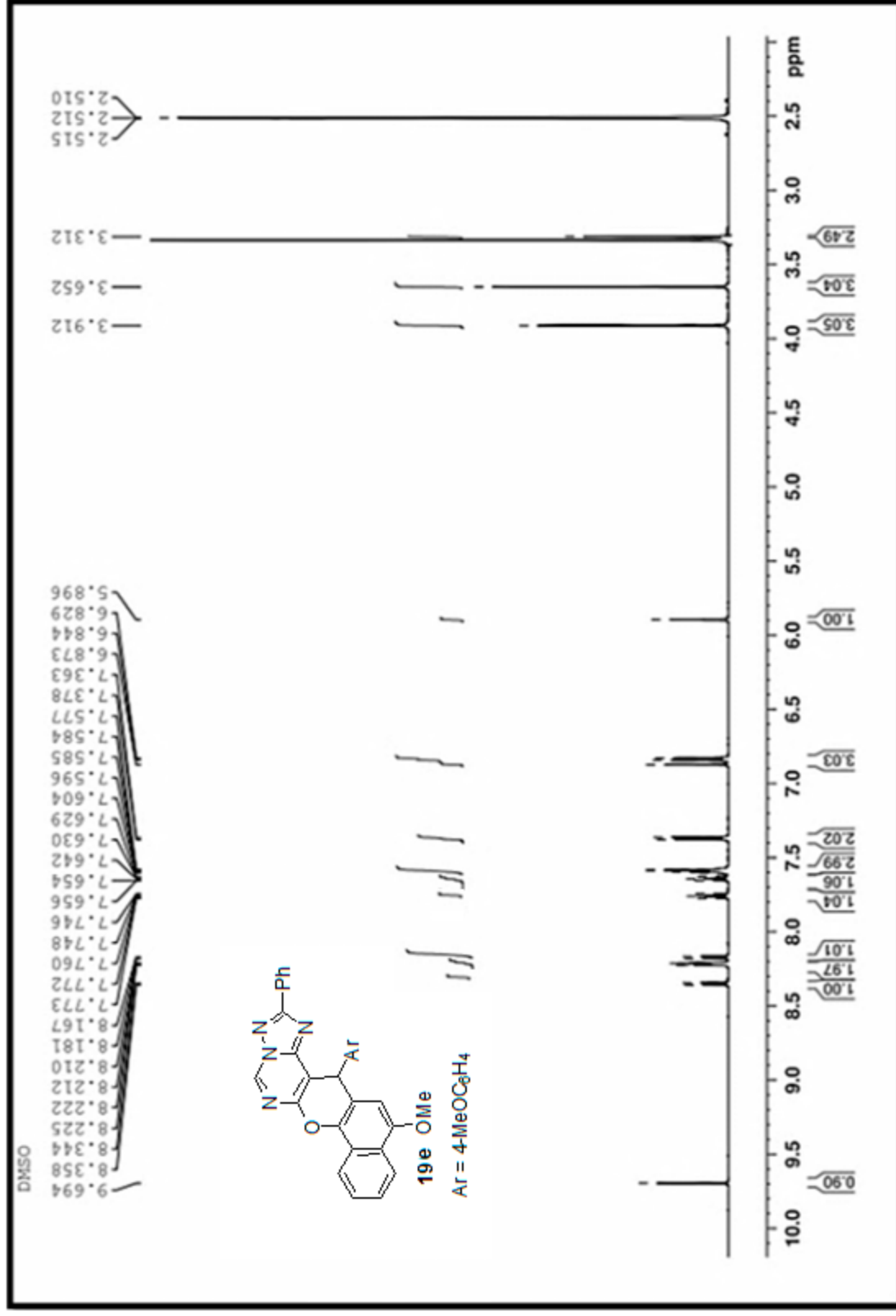

Supplement: Supplementary file 1 [file molecules-22-00479-s001.zip › molecules-178589-supplementary/1H NMR of compound 19e.pdf]
