# Supplementary material for: Design of New Benzo[h]chromene Derivatives: Antitumor Activities and Structure-Activity Relationships of the 2,3-Positions and Fused Rings at the 2,3-Positions
Source: Molecules. 2017 Mar 18;22(3):479. doi: 10.3390/molecules22030479 (PMC6155235; doi:10.3390/molecules22030479)
Supplement: Supplementary file 1 [file 13C NMR of compound 6.pdf › molecules-178589-supplementary/13C NMR of compound 6.pdf]

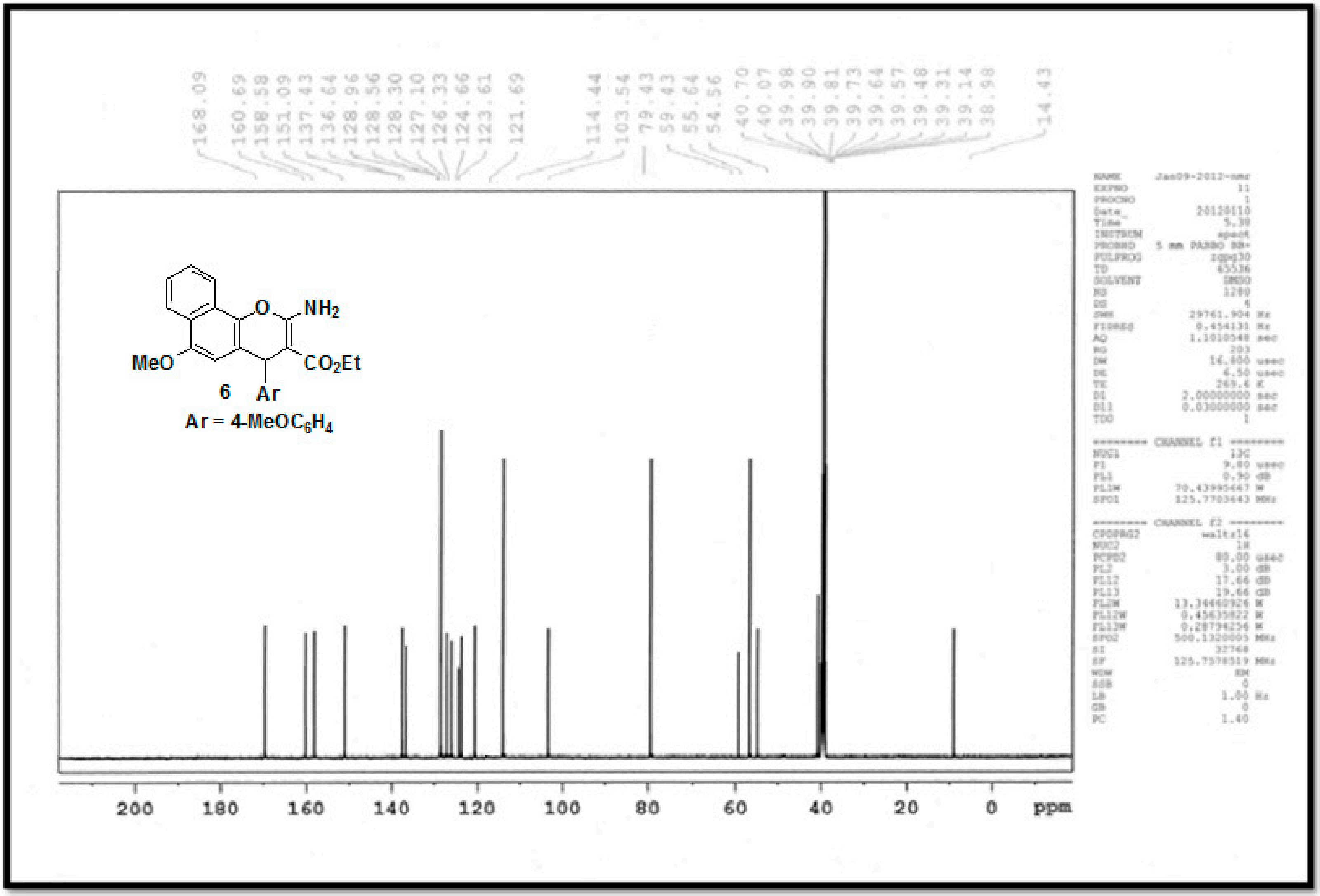
168.09
160.69
158.58
151.09
137.43
136.64
128.96
128.56
128.30
127.10
126.33
124.66
123.61
121.69
114.44
103.54
79.43
59.43
55.64
54.56
40.70
40.07
39.98
39.90
39.81
39.73
39.64
39.57
39.48
39.31
39.14
38.98
14.43
MeO
O
NH2
CO2Et
6
Ar
Ar = 4-MeOC6H4
200
180
160
140
120
100
80
60
40
20
0
ppm
